# Additive Manufacturing of Polyhydroxyalkanoate-Based Blends Using Fused Deposition Modelling for the Development of Biomedical Devices

**DOI:** 10.3390/jfb14010040

**Published:** 2023-01-10

**Authors:** David Alexander Gregory, Annabelle T. R. Fricker, Peter Mitrev, Meghna Ray, Emmanuel Asare, Daniel Sim, Soponvit Larpnimitchai, Zixuan Zhang, Jinge Ma, Santosh S. V. Tetali, Ipsita Roy

**Affiliations:** Department of Materials Science and Engineering, Faculty of Engineering, University of Sheffield, Sheffield S10 2TN, UK

**Keywords:** Polyhydroxyalkanoates, thermoplastic polymers, 3D printing, tissue engineering, biomedical devices, regenerative medicine

## Abstract

In the last few decades Additive Manufacturing has advanced and is becoming important for biomedical applications. In this study we look at a variety of biomedical devices including, bone implants, tooth implants, osteochondral tissue repair patches, general tissue repair patches, nerve guidance conduits (NGCs) and coronary artery stents to which fused deposition modelling (FDM) can be applied. We have proposed CAD designs for these devices and employed a cost-effective 3D printer to fabricate proof-of-concept prototypes. We highlight issues with current CAD design and slicing and suggest optimisations of more complex designs targeted towards biomedical applications. We demonstrate the ability to print patient specific implants from real CT scans and reconstruct missing structures by means of mirroring and mesh mixing. A blend of Polyhydroxyalkanoates (PHAs), a family of biocompatible and bioresorbable natural polymers and Poly(L-lactic acid) (PLLA), a known bioresorbable medical polymer is used. Our characterisation of the PLA/PHA filament suggest that its tensile properties might be useful to applications such as stents, NGCs, and bone scaffolds. In addition to this, the proof-of-concept work for other applications shows that FDM is very useful for a large variety of other soft tissue applications, however other more elastomeric MCL-PHAs need to be used.

## 1. Introduction

A key challenge in modern healthcare is the ability to provide patient specific bespoke treatment. In addition to this, tissue engineering scaffolds for biomedical applications require increasingly more complex designs in an effort to mimic natural tissues. This includes both the materials used as well as the physical architecture of the scaffold structures that harbour the cells. The goal is to replace damaged or diseased tissue, bones and organs with patient compatible working implants that do not exhibit any immune response and are gradually fully integrated into the body, resulting in a complete recovery of the diseased area. Here, we aim to use an advanced manufacturing (AM) approach, namely Fused Deposition Modelling (FDM) to produce complex structures in 3D for a large variety of important biomedical applications including tissue repair patches (e.g., cardiac patches), bone repair, tooth implants, osteochondral tissue repair, nerve guidance conduits, coronary artery stents and heart ventricles. 

Although 3D printing technologies are becoming more and more popular, there are still many limitations to be overcome. Many issues revolve around the availability of a suitable material as well as important essential properties including biocompatibility, biodegradability, processability and suitable mechanical properties. To date most research exploiting 3D printing technologies has revolved around polylactic acid (PLA) [1], poly(DL) lactic acid (PDLLA) [2], poly(L-lactic acid) (PLLA) [3,4], poly(lactic acid-co-glycolic acid) (PLGA) [5,6] and polycaprolactone (PCL) [7] amongst other thermoplastic polymers. However, very little research has focused on the 3D printing of Polyhydroxyalkanoates (PHAs). Given their sustainability, biodegradability and biocompatibility, commercial companies all over the world are becoming increasingly interested in the use of these polymers for many, vastly different applications. The FDA approval of PHAs (P(4HB)) for use as sutures by TephaFLEX^®^ and the PHASIX™ plug and patch used in the repair of inguinal hernias [8] confirms their biocompatibility and has established their usefulness for biomedical applications. This increased interest in PHAs has led to the recent commercial development and production of PLA/PHA blend filaments for popular extrusion filament printers from companies such as ColorFabb (PLA/PHA) and 3DPrintLife (PLAyPHAb^TM^). Therefore, in this work we have designed and printed 3D scaffolds with the ColorFabb “natural” (PLA/PHA) filament as proof-of-concept work for a range of biomedical applications, which we believe will illustrate the huge potential of 3D printing of biocompatible thermoplastic polymers and in particular the PHA/PLLA blends. In the following sections we briefly introduce PHAs, the overall 3D printing methodology along with background for the various applications investigated in this study. 

### 1.1. Polyhydroxyalkanoates, Novel Biopolymers for 3D Printing

Polyhydroxyalkanoates (PHAs) belong to a large family of biopolymers, which are accumulated as the internal reserves of carbon and energy within some PHA producing bacteria such as *Pseudomonas mendocina*, *Bacillus subtilis*, *Cuprivadus necator* and *Alcaligenes latus* [9,10]. PHAs are produced via bacterial fermentation under stressed growth conditions such as an excess in carbon and a limitation of nitrogen [11]. PHAs are noted for their diverse structures and wide-ranging material properties, which are determined by the number of carbon atoms present in their monomeric units. PHAs are classified into Short-chain length (SCL) PHAs, 3 to 5 carbon atoms in their monomer units and medium chain length (MCL) PHAs, with 6 to 16 carbon atoms in their monomeric units [9]. SCL-PHAs are generally known to be hard and brittle with high melting temperatures, high crystallinity and lower elongation at break values, whereas MCL-PHAs are characterized as highly elastomeric, with lower melting and glass transition temperatures [9]. 

In addition to this, the properties of PHAs can be tuned to meet the requirements of specific applications. This can be done by either blending two PHAs with different characteristics or by varying fermentation parameters such as substrate feed (e.g., fatty acids) to form copolymers [12]. Due to their biological origin, PHAs degrade under physiological conditions via surface erosion into natural metabolites such as 3-hydroxybutyrate and other hydroxyacyl-CoAs. Therefore, PHAs have been reported to exhibit non-immunogenicity and excellent resorbability that allow them to be easily incorporated into biological systems [9,12]. These features, coupled with their desirable structural diversity, make PHAs ideal materials for many biomedical applications. Bioresorption via surface erosion ensures the maintenance of the stability of the implant in line with the rate of tissue repair and regeneration. 

The biocompatibility of PHAs has been widely shown through in vitro studies with a large range of cell types, as well as in many different animal models for ex vivo and in vivo studies. Poly(4-hydroxybutyrate) gained FDA approval for use as a suture material in 2007 [13], and has since been approved for other medical applications in the USA and across Europe [14]. The devices sold for clinical use include surgical films, meshes, plugs and patches, and are used for applications such as for the repair of inguinal hernias. PHAs are also being researched for a large number of further biomedical applications [15], including tissue repair patches [16], cardiac patches [17], cardiac valves [9,18], stents [19], nerve guide conduits [20], cartilage [21] and bone scaffolds [22], and drug carriers [23].

### 1.2. Fused Deposition Modelling

**Figure 1:**
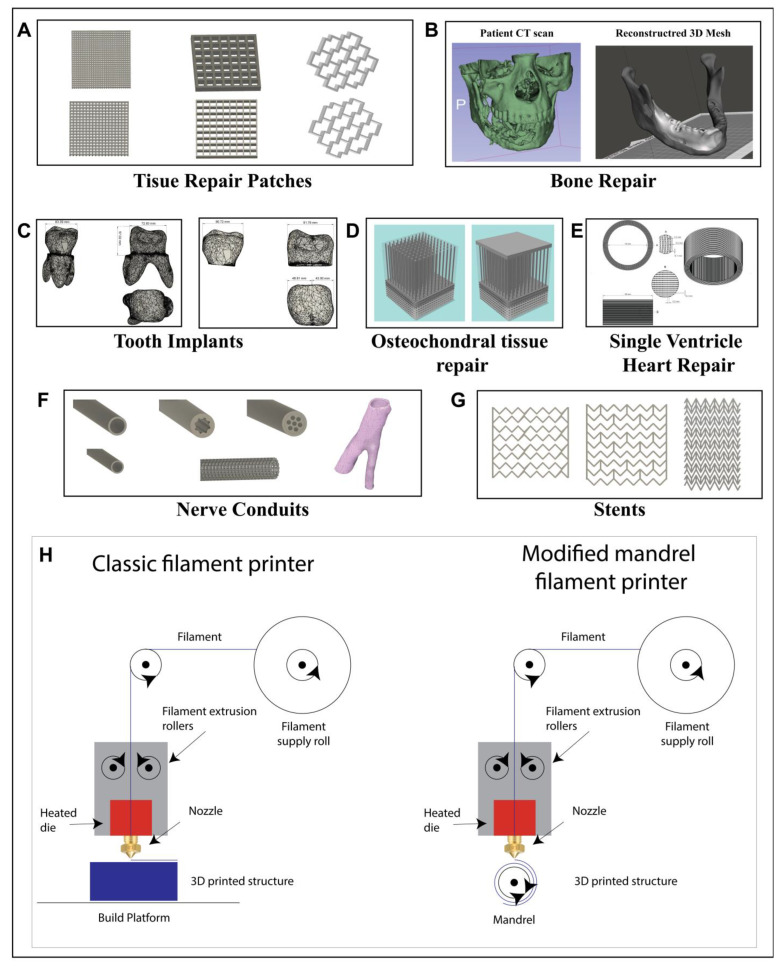
Overview Figure showing all the designed structures, (**A**) The grid, woodpile and accordion structures for use as tissue healing patches e.g., cardiac tissue; (**B**) Bone implants from patient CT scans e.g., repair of the damaged mandible; (**C**) Tooth implants from patient CT scan data; (**D**) Osteochondral tissue repair plug implant, (**E**) Single Ventricle Heart repair conduit, designed as a mesh to be permeable; (**F**) a variety of nerve guidance conduits, right example of a CT scan mesh of a human sciatic nerve, demonstrating the ability to repair complex nerve junctions; (**G**) Variety of different polymer stent designs (flattened out). (**H**) Schematic illustration depicting the filament extrusion process for a classic filament printer (**left**) and our modified mandrel printing (**right**).

Fused deposition modelling (FDM) is probably one of the most well-known types of 3D printing in the Additive Manufacturing (AM) industry. The main reason for this is the widespread availability of cost-effective benchtop printing systems such as Creality’s Ender and the Ultimaker 3D printer series. Most AM printing systems employ a similar methodology of creating 3D structures. Initially, 3D models are generated via a computer aided design (CAD) software package. Industry standards of these are Autodesk Inventor, Fusion 360 and SOLIDWORKS. Once the model has been made it is commonly exported as an *.stl file format. This is a widely accepted format that can be post-processed with almost all currently available slicing software packages. For printing purposes, the model must be sliced into layers, which are then deposited layer by layer (LBL) on top of each other during the printing process. The layer thickness needs to be selected according to the material used, printing temperature and nozzle diameter. Here the selected slicing software used were Creality Ender slicer powered by the Cura engine and Ultimaker Cura 4.9.1, however there are many more slicing software available. The slicing software then converts the stl model file into g-code, which describes exactly line by line how the printer moves the printhead and how much material is deposited in predefined locations. Most commonly FDM printers use a filament-based system where the filament is fed via a motor through a heated nozzle (Bowden filament-fed extruder, Figure 1H (left)). Other extruder variants exist including screw fed extruders and syringe extruders with mechanically or pneumatically driven plungers, these are ideal for printing powders and pellets [24]. In this work, we use a filament-based Bowden extrusion system. 

### 1.3. Printing of PHAs

As previously described, at present very little work has been done regarding the printing of PHAs. Our group is a key contributor to the pioneering work of 3D printing PHAs, however to date not much has been published in this emerging area. A very limited number of studies exist that use commercially available filaments for the printing of PLA/PHA structures. However, these do not discuss the use of these in biomedical applications [25]. Further to this there are some studies that have investigated the production of PHA composite filaments [26,27], again little research has been done printing these filaments. As far as other printing technologies go there have been a some reported studies using selective laser sintering (SLS) for the printing of PHBV and Ca-P/PHBV bone tissue scaffolds [22] and Computer-aided wet-spinning (CAWS) printing P(3HB-*co*-3HHx) lattice designs for use in a New Zealand rabbit radius model, investigating optimal bone regeneration conditions [28]. 

### 1.4. Introduction of Applications

The following will introduce the various biomedical applications considered in this study with basic background information, for which FDM can be applied to help enhance prototype development and produce patient specific implants and scaffolds to help solve many medical challenges targeted towards custom designed healthcare. 

#### 1.4.1. Tissue Repair Patches

Tissue repair patches include engineered constructs that can, for example, aid in wound healing, and act as a carrier for the delivery of cells to an area of damaged tissue. The healing of injured tissue can be encouraged, for example by using electrostimulation to affect cell behaviour [29], anti-inflammatory agents [30], or by functioning as an efficient drug delivery system for the control of infection [31]. Past and current research into wound healing patches use synthetic polymers such as polyvinyl alcohol (PVA) [31] and polycaprolactone (PCL) [30], and natural materials such as sodium alginate [31], gelatin and bacterial cellulose [32,33]. Tissue repair patches are also being researched for use in the repair of congenital heart defects [34], soft tissues such as abdominal wall [35], and inguinal hernia patches [36]. 

The generation of tissue repair patches is one of key importance for the biomedical field when treating patients. One area of significant importance is cardiovascular diseases (CVDs). CVDs are the leading cause of deaths worldwide [18,37], accounting for around 17.9 million per year according to the World Health Organisation (WHO), and many lead to myocardial infarction (MI). Developing treatments that can aid in the regeneration of this area of scar tissue are vital to reducing post-MI mortality.

At present suggested patches have been made from a wide range of materials, for example omentum [38], decellularised myocardial tissue [39], poly(glycerol sebacate) (PGS) [40], gelatin/hyaluronic acid [41], fibrin [42], poly(lactic acid) (PLA)/poly(ethylene glycol) (PEG) blends [43], and in recent research Polyhydroxyalkanoates (PHAs), in particular poly(3-hydroxyoctanoate), P(3HO), have been investigated [16]. 

Cardiac patches have been produced via a variety of processing techniques, including solvent cast films [16], excimer laser micro-ablation [44], thermally induced phase separation [45], electrospinning [17], and 3D printing [41]. In this study a PLA/PHA blend will be used as a proof of concept for a 3D printing processing technique for the production of cardiac patches amongst other biomedical devices.

#### 1.4.2. Bone Implants

Every year many people suffer from broken bones and limbs, with rates of 3.6 fractures per 100 people in England (2008), as well as those who are born with defects and suffer from illnesses such as bone cancer that cause them to have tissue removed and replaced. This results in the need for many different medical bone implant designs, of varying sizes and forms. State-of-the art procedures used currently to repair and reconstruct bone rely on bone grafts from the patient, titanium implants and plates [46]. Many of today’s procedures were originally developed in the first world war and have not evolved since. They are inefficient and require long surgery times, for example one such procedure is mandibular reconstructive surgery. This requires a tibial graft be to removed, shaped, and then fixed in a new location using plates. This causes these techniques to be crude and have a high failure rate in preserving the individual patients’ anatomy. Furthermore, procedures involving total replacement using titanium implants, such as a hip replacement, still rely on batch machined implants with discrete sizes that fail to accommodate each patient’s unique physiology.

The complexity and difficulty associated with the machining method of manufacturing requires implants to be produced in batches and has presented a major challenge in the development of bespoke implants. However, with the recent development of the disruptive 3D printing technologies these challenges are overcome as the patients’ individual anatomy and even internal anatomy, such as nerve channels (e.g., inferior alveolar nerve for a mandible is presented in this paper), can now be preserved. There have already been studies conducted into the 3D printing of titanium hip implants using SLS, SLA and 3D printed facial structures via FDM techniques [47].

Hip implants are one of the most common implant procedures in modern medicine and continue to grow because of an increasingly ageing population. Wang et al. investigated the performance of titanium 3D printed hip implants [48]. Out of 74 patients, 57 received a conventional hip replacement, with the remaining 17 receiving 3D printed hip replacements. The 3D printed hip replacements were designed using CT scans of the patient’s hip joint to model a suitable patient-specific replacement. Once the implant had been designed it was 3D printed using powder bed selective laser sintering (SLS). This process involves the use of a high-powered laser to melt titanium powder together to form the implant. The study showed that the 3D printed replacements resulted in a higher Harris score (questionnaire used by doctors to determine performance of implant via assessing the patient’s pain and function) and patients were able to walk and apply weight to the hip sooner than the patients who received the conventional implant. 

In contrast to novel 3D printing, the current state of the art for surgical implants is to replace or reconstruct lost tissue via the use of a tissue graft, a bioinert implant, or a bioresorbable implant. For example, Ow et al. (2016) used a titanium implant in order to replace a portion of a patient’s mandible that had been removed due to a tumour [49]. The patient suffered from ameloblastoma and had to undergo a partial mandibulectomy to remove the diseased tissue. This procedure left the patient with a large portion of their jaw missing. To restore facial features and function to the patient’s jaw a custom-made implant was designed using CT scans and modelling software. The replacement model was designed with the intent of preserving the original form of the patient’s mandible. This was then machined from titanium and the implant fixed using six screws into the healthy mandible, restoring the patient’s facial anatomy [49]. This is an example of a top-down approach where a block of metal is used, and excess metal is machined away according to the CAD design. This process is both wasteful, time consuming, and requires expensive CNC machinery. However, the advantages of using CT images to produce patient specific implants with ideal dimensions suitable for the repair, strongly demonstrated the advantages of driving research in this direction. 

To build on the success of creating patient specific implants via CT images, in this work, we demonstrate the use of real patient CT scan data and create implants that directly replicate and copy the patient’s anatomy to construct an anatomically accurate implant. Furthermore, in contrast to top-down approaches, for a mandible, the ability of reproducing an implant that can accommodate nerve channels is not possible due to processing limitations. To our knowledge, the fabrication of a mandible implant, which includes a hollow channel to accommodate the nerve channels within the 3D structure has not been implemented to date. Hence, in this work, for the first time we have included the nerve channel within our implant and we believe this will enable better integration into the patient for next generation bioresorbable implants. Further to this we demonstrate this with biodegradable natural polymers PLA/PHA. This proof-of-concept work aims to illustrate the accurate 3D printing of the bone scaffold constructs which in future can be seeded with stem cells such as bone marrow mesenchymal stem cells [50,51,52] for bone regeneration and eventual total replacement of the implant. 

#### 1.4.3. Tooth Implants 

Another important area in the field of biomedical applications is the need for the repair of teeth including the development of tooth implants. Edentulous patients suffer from several consequences, including obvious ones such as missing teeth causing difficulties with chewing. Furthermore, other effects such as the collapse of the maxillofacial region and inadvertently affecting their appearance give rise to mental health issues [53]. Unlike other parts of the body, teeth have very limited regeneration capability, due to a combination of tooth anatomy changes due to aging, affecting pulp regeneration and the patient’s oral environment. This therefore means that adult patients with tooth loss are not able to rely on their own self-repair functions to regenerate their teeth. 

In order to meet the normal chewing requirements for edentulous patients, three treatment choices can be considered [54]: The first is the use of removable partial dentures which can only restore the chewing function partly; The second is the use of artificial implants supporting fixed partial dentures, but this type of treatment requires a large number of implants and is costly and painful; The third method is extracting the remaining healthy teeth and providing a complete denture or overdenture supported by the implant, which might need bone reconstruction. In this study we consider the second option to produce biocompatible artificial implants to replace missing teeth [55].

For this the resultant tooth replacement needs be a mechanically sound restoration that withstands occlusal forces, preserving tooth structure, ensuring top and bottom teeth match upon closing the mouth, and also provide an aesthetic restoration preferably unnoticeable from the original teeth [53]. Currently the accepted materials, including metals, ceramics and polymers, exhibit low cell viability and limited vascularization [56]. 

Furthermore, when designing tooth implants it is important to take the tooth structure into account, for example the molar teeth are made up of two main structures: a crown (the section over the gum line) and the roots (the section below the gum line) [57]. This therefore means the materials for these two sections of the tooth should be considered separately as their mechanical properties also vary significantly. The root should allow nutrients to diffuse into transplanted tissue/cells and guide cells into the correct orientation and structures [56]. A potential candidate for this could be P(3HB), as this has been widely used in clinical applications [58]. In contrast, the crown of a tooth needs to be stable enough to withstand the force and wear associated with long term chewing, and therefore a non-biodegradable material with high toughness, sufficient rigidity, good thermal stability, and high resistance to chemical attack and environmental stress and cracking is required [59,60]. Here a thermoplastic polymer such as acrylonitrile butadiene styrene (ABS) could be considered as a potential candidate. 

As discussed in the previous section (Section 1.4.2) 3D imaging technologies such as CT, ultrasound imaging, and magnetic resonance imaging (MRI) techniques are being used to create 3D models of real structures in patients in order to produce more structurally authentic and patient-specific implants within micrometre accuracy [61]. The current state-of-the-art method is also the use of expensive production of implants via CNC machining. This however requires time consuming procedures to ensure that suitable implants are fabricated that perfectly fit the patient, leading to an expensive process [62]. The complexity of the tooth structures makes 3D printing a desirable technique for next generation tooth implants [63]. 

#### 1.4.4. Osteochondral Tissue Repair

Degenerative diseases such as osteoarthrosis and traumatic injuries to joints can damage the integrity of the cartilage within the synovial joints and lead to a progressive loss of function, severe inflammation, and increasingly drastic lifestyle changes for the patient [64]. 

The synovial joints of the human body, e.g., fingers, elbows, shoulders, hips, knees and ankles, are a complex system with the intent of connecting two hard, cancellous bones together and allowing an efficient and non-abrasive transfer of energy. The overall shape of these joints varies by location and desired functional movement; however, all the joints are characterized by the ends of the bones within the joint being capped with a critical layer of cartilage. This cartilage is known as articular cartilage and is mostly comprised of Type II collagen, designed in an intricate and specifically oriented geometry with the bone it transitions from, and filled with water to give its characteristic high compression modulus [65,66].

The degradation of articular cartilage tissue causes the advancement of the tidemark and mineralized cartilage, lowering the efficacy of the joint and causing inflammation, pain, and stiffness, resulting in loss of both function and endogenous tissues within the joint [67,68,69]—this is also known as arthrosis. 

There are many known risk factors for arthrosis including genetic pre-disposition, obesity, type of physical activity and the levels of intensity and incidence, and congenital or developmental conditions such as hip dysplasia [70,71]. In addition to this, age is a key factor, and for people over the age of 60 the probability incidence of arthrosis is over 80%. This causes a major economic burden on healthcare systems around the world [64,72]. 

Current treatment strategies range from simple lifestyle changes to avoid excess force and stress upon the damaged joint for early-stage degeneration or very small defects in the tissue [73]. Where pain becomes overwhelming, a common treatment is to surgically induce joint ossification between the two bones, known as arthrodesis, and this is most commonly for joints in e.g., the spine, hands, and feet. For major joints such as hips, the surgical treatments involved become more complicated and extensive in remodelling and/or reconstructing the joint with implants and biomaterials [74]. Current treatment strategies transition from more conservative methods with simple lifestyle changes to avoid excess force and stress upon the damaged joint for early-stage degeneration or very small defects in the tissue [73].

As many of the traditional methods of treatment for arthritis are far from ideal and often do not result in full recovery, there is much research aimed at restoring the defective or missing cartilage tissue with regenerated replacement tissue. Here, the use of 3D scaffold designs for cartilage tissue aimed to mimic the endogenous different tissue stages have great potential. Chondrocyte growth is dependent on the use of three-dimensional tissue scaffold environments [75,76]. Current research into 3D tissue scaffolds for repairing or replacing the osteochondral zone is very much in its infancy and therefore much research is needed, this includes the materials used as well as fabrication methods and structure. Here, we propose an FDM 3D printed construct made from PLA/PHA, where the printability of the construct together with printing resolution are investigated as a proof-of-concept study. 

#### 1.4.5. Peripheral Nerve Injury and Nerve Repair

Peripheral nerve injury (PNI) is defined as the degeneration of damaged axons, which leads to permanent functional defects [77]. Several factors including trauma, birth injury, bone dysplasia, and entrapment neuropathies, among others may cause PNI [78]. These are often associated with disruption or complete defect of sensory and/or motor function in the areas associated with the injured nerve [79]. Nerve injuries can have severe consequences on the patient’s life quality due to potential paralysis of the affected limbs as well as neuropathic pain [80]. Recent studies have shown that the proportion of PNIs among trauma patients has increased to approximately 2.8% of which more than 50% patients failed to recover normal motor and sensory functions following treatment [81].

Although, neurons in the peripheral nervous system (PNS) have shown intrinsic plasticity to regenerate and form functional connections with their targets, if the damage is severe, full repair might not occur without external intervention and treatment [82,83]. PNI with gaps of less than 5 mm can naturally regenerate, however, nerve gaps larger than 5 mm often present poor axonal regeneration which leads to incomplete functional recovery [84,85]. Peripheral nerve regeneration occurs after the cascades of Wallerian degeneration [86]. To date, no therapeutic method has been developed to facilitate the rate of peripheral nerve regeneration [77]. Despite efforts, full recovery of motor and/or sensory function after severe PNI is yet to be achieved [87]. The current ‘gold standard’ treatment strategies, which have proven effective for non-critical gap injuries, are end-to-end suturing and nerve grafts. These treatments however have substantial disadvantages for larger injury gaps. End-to-end suturing is only viable for injury gaps up to 10 mm as long as this does not result in tension on the nerve which could otherwise impair blood flow. More severe nerve injuries of up to 30 mm gaps have reportedly been repaired successfully by means of nerve tissue grafts, however here the biggest limitation is access to donor tissue [81,88]. Meta-analysis of data indicates that only 51.6% patients achieve satisfactory motor recovery and 42.6% experience satisfactory sensory recovery from median and ulnar nerve repairs [89]. 

In view of these drawbacks, scientists have been developing nerve guidance conduits (NGCs), which are designed to fully repair and restore nerve functions of larger nerve injury gaps without the need for donor tissue. A Nerve Guidance Conduit (NGC) is a tube-like bridge interposed between the two stumps of the injured nerve which guide the regenerating axons from the proximal end of the injured nerve to reconnect with the distal end and restore full function [90]. To date it has been reported that nerve defects have been repaired both experimentally and in clinical practice by bridging the gap with NGCs (tubulization) [91]. Even though, NGCs have demonstrated the potential of restoring optimal function following injury, their current efficacy is limited to injury gaps of 30–40 mm and their performance is only comparable to autografts for gaps of up to 10 mm. 

NGCs have been fabricated from both synthetic materials like silicone and natural polymers (e.g., PHAs). The most primitive NGCs are simple hollow tubes. Examples of these made from silicone exhibit low biocompatibility and potential of fibrotic encapsulation, furthermore due to their non-resorbable and brittle nature they require a second surgery to remove their remnants, once the nerve has reconnected [92,93]. This is the reason attention has shifted toward natural, biodegradable, bioresorbable and biocompatible polymers for the fabrication of third generation NGCs [94]. Amongst these, PHAs have been reported to exhibit exceptionally suitable properties for NGCs [20,95,96,97]. As is often the case for surgical procedures every injury and person are different and therefore the nerve conduit should be of correct dimensions for the site of interest. Here 3D printing presents itself as a natural choice of fabrication that can quickly and efficiently 3D-print complex designs at the correct dimensions. It has been previously reported by Zeng et al. (2014) and Liu et al. (2018) that multi-channel PLLA NGCs promoted differentiation of neural stem cells (NSCs) into neurons [98,99]. Therefore, amongst others we propose a variety of NGC designs including multichannel solutions printed with the PLA/PHA material blend. We demonstrate the ability to precisely and accurately produce intricate designs that are suggested to be beneficial for nerve regeneration.

#### 1.4.6. Stents

Cardiovascular disease has become one of the most common reasons of death all over the globe. In 2016, coronary artery disease (CAD) caused around 9.43 million of deaths. According to the prediction of American Heart Association, in 2035, the direct and indirect costs, loss of work hours by cardiovascular disease (CVD) will lead to a total cost of $1.1 trillion. Thus, the use of stents to manage CVD is an urgent medical need [100]. Percutaneous transluminal coronary angioplasty (PTCA) has become the state of the art treatment for coronary artery disease [101]. However, a key response in the human body is restenosis and thus limits the benefit of stents [101]. Restenosis is when an artery or valve becomes abnormally narrow again after corrective surgery. Clinical and animal experiments indicate that restenosis in the internal vessel wall after coronary intervention is caused by a combined effect of neointimal formation and geometric remodelling [101]. There are two effective strategies to avoid restenosis, inhibition of neointimal formation by the use of antiproliferative agents, such as the protein tyrosine kinase inhibitor ST638, and prevention of geometric remodelling by the application of stents [101]. The design of the coronary stent impacts the distribution of wall shear stress which in turn affects the advancement of endothelialisation, neointimal hyperplasia, and subsequent restenosis [102]. First-generation stents were fabricated from metal, these include the S7 and NIR stents designed by Medtronic AVE (Marlborough, MN, USA) and Boston Scientific (Marlborough, MA, USA), respectively. The diameter of the aforementioned are 3.5 mm, which is similar to the diameter of large human and animal blood vessels [103]. The strut thicknesses of the metal stents are in general 0.1 mm. The wave pattern of S7 led to less vascular injury than the grid-like cells of NIR [103]. Beier et al. investigated two commercial stents, “Omega” (Boston Scientific, Marlborough, MA, USA) and “Biomatrix Flex” (Biosensors International, Wilmington, DE, USA). Their design used 4 mm as the diameter and 0.081 mm and 0.12 mm as strut sizes respectively [104].The mean strut spacing of Omega is 1.4 mm and 1.6 mm for Biomatrix. According to their study, the ideal intra-strut angle is around 40° [104]. Analysis by mathematical modelling gave that the optimal intra-strut angle of the metallic grid stent to be between 38.5° and 46° [102,105]. Hara and his group found thinner struts performed better in preventing restenosis than the thicker ones and the thicknesses in their investigation were 0.05 mm as the thinner strut and 0.14 mm as the thicker strut size [106]. 

Bare metal stents were initially used to treat coronary artery disease, but this treatment led to a risk of in stent restenosis and the need for target lesion revascularisation. The evolution of drug eluting stents seemed to initially reduce the complications and became standard in clinical practice for coronary artery disease. There is growing body of data that shows that drug eluting stents can result in very late stent thrombosis and intimal hyperplasia to different degrees i.e., in stent restenosis. Thus, polymer stents have become a focus of study and are expected to replace metallic stents [107]. One of the best known biodegradable stents currently available is the Igaki-Tamai stent (Igaki Medical Planning Company, Kyoto City, Kyoto, Japan) made using PLLA [108]. This stent has been used both for coronary artery and peripheral vascular disease. A 10-year follow-up study showed that the cause of death from any cause was 13%, with 2% mortality from cardiac causes and 50% having major cardiac events [109].

#### 1.4.7. Single Ventricle Condition of the Heart/Coronary Artery Repair 

A single ventricle condition is where there is only one functional ventricle of the heart present, the most common example being Hypoplastic Left Heart Syndrome (HLHS). Patients with HLHS suffer from a poorly developed heart, with the most significant effect being the underdevelopment of the left ventricle. The effect of a weak left ventricular wall leads to poor support for systemic circulation, as there is not sufficient muscle to eject the blood with adequate force to exit the heart and reach the extremities [110,111]. There is also a mixing of the oxygenated and deoxygenated blood in a hypoplastic heart due to the pulmonary and systemic circulations being in parallel as opposed to in series, resulting in what is known as a cyanotic heart defect. These defects are characterised by a desaturation of arterial blood, and consequently provide inadequate oxygen delivery to the tissues [112,113].

The gold standard treatment of HLHS involves a series of three staged surgical procedures with the desired end result being a redirection of venous blood flow towards the pulmonary circulation for oxygenation, as occurs in the normal heart [114,115], along with causing the existing right ventricle to take on the usual role of the left in the maintenance of systemic circulation. The construct described in this work is designed to have its use in the final stage of these three operations, known as the Fontan procedure. This intervention involves the insertion of a conduit to form a connection between the inferior vena cava and pulmonary artery, which is also known as a total cavopulmonary connection [110]. This acts to redirect the blood flow so that oxygenation can occur prior to distribution around the body, and also gives a provisional left ventricle to replace the existing underdeveloped structure [114]. The three central aims of the Fontan procedure can therefore be identified as relief of cyanosis in systemic circulation, maintenance of cardiac output, and reduction in the volume loading of the systemic ventricle (the right ventricle) [116].

Currently, majority of the conduits used for the Fontan procedure are composed of expanded poly(tetrafluoroethylene) (ePTFE); commercially sold under the name Gore-Tex^®^ or Teflon. This is a thermoplastic, fluorinated polymer with good mechanical integrity [117]. It has been described as a biostable polymer due to its resistance to degradation in biological environments, and it is also antithrombotic, making it an ideal material for a long term cardiovascular and blood contacting device [118]. However, the use of ePTFE comes with the limitation of a lack of growth potential. This means that as the patient grows the conduit will stretch, bringing a risk of stenosis or narrowing of its lumen [119,120]. For this reason, many patients require revision surgeries when they outgrow their conduit, or otherwise often suffer from the consequences of a stenosis of their graft. This demonstrates the vital need to develop a conduit that encourages cell growth and attachment, as well as exhibits a controlled biodegradability, giving the construct future growth potential and total integration into the body. 

Further to this, another limitation of current approaches to the development of conduits for this procedure is their lack of contractility. This means that they are unable to generate a pressure higher than that of the pulmonary vasculature. This pressure gradient is the opposite to what is normally found in the cardiovascular system and is the cause of many complications associated with this procedure. A lower pressure within the conduit compared to that in the pulmonary vasculature results in increased pulmonary resistance, as blood is flowing against its pressure gradient. The lack of contractility also means there is no propulsive force to assist in pushing the blood against this pressure gradient. Sequalae of this effect includes those such as pulmonary vascular remodelling, refractory ascites and pleural effusions [121,122]. 

Contractility of the conduit is also important in maintenance of the laminar flow of blood. Stasis of blood flow is a known risk factor for thromboembolic disease, which is another mechanism in which stenosis of the graft may occur [123]. With coordinated contraction of the conduit this gives simultaneous propulsion of the blood in one direction, maintaining its directionality and therefore laminar flow. A lack of contractility has also been associated with endothelial dysfunction in the conduit and can contribute to incorrect vascular remodelling in the pulmonary system, and as a result contribute to the increased pulmonary vascular resistance [124].

Therefore, the ideal implant would act as both a temporary conduit for the redirection of blood within a single ventricle heart and as a scaffold to guide the generation of a pulsatile tube of cardiac tissue which will act as a provisional left ventricle. This should be able to contract with sufficient force and be able to give a pressure of 5–6 mmHg above that of the pulmonary circulation, therefore re-establishing the pressure gradient normally seen in the cardiovascular system. Here we propose a proof-of-concept porous scaffold design as a basis for a contractible tube printed via FDM.

In this work we will investigate, for the first time, this array of biomedical examples, demonstrating the potential possibilities of employing FDM of biodegradable thermoplastic polymers as tools to move towards a new state of the art solutions for patient specific care and successful recovery. 

## 2. Experimental Methods

### 2.1. General Preparation of 3D CAD Model Files

Two main methods were used to generate models for the applications discussed in this work. The first method was using Autodesk Fusion 360 or Autodesk Inventor 2020 to directly draw the CAD files with researched dimensions believed to be most suitable for each application (described in more detail for each application). This includes tissue repair patches (A), osteochondral tissue repair (D), single ventricle heart repair (E), stents (G) as well as some of the nerve guidance conduits (F) (see Figure 1). During the modelling process it was important to consider the nozzle diameter that was being used, as the models were designed in such a way that the features were the diameter of the nozzle rather than simply designing a block and then using infill parameters to design the fine features. This allowed us to get maximum flexibility in terms of model design and the direction in which the printhead would move.

The second method was to obtain real patient computer tomography (CT) imagery data and extract features based on their material density in form of 3D mesh data. Here we extracted bone, tooth and nerve tissue data by altering tissue density thresholds to isolate the areas of interest. After extracting the 3D mesh data, the structure was then further reconstructed using Autodesk Mesh Mixer (Version 11.4.35). This was to remove artifacts and holes in the models which would cause errors in the post processing of the models during slicing. 

Both methods were then used to create the commonly used *.stl (stereolithography) 3D printing file format. The files were then sliced in Creality Slicer 1.2.3 (powered by the Cura engine) or Ultimaker Cura 4.9.1 and printed with a Creality Ender 5 FDM printer (see schematic of the printing process Figure 1H) using the commercially available ColorFabb PLA/PHA natural filament. Depending on the model design different nozzle sizes ranging from 0.15, 0.2, 0.3 and 0.4 mm were used with a general extrusion temperature of 205–215 °C. 

For the single ventricle flat model design a custom built 8 mm cylindrical mandrel was designed and connected to the 3D printer prior to the printing to be able to print a 3D meshed tube structure, Figure 1H (right). This was then calibrated, and calibration values were edited directly into the g code to ensure distances were correctly represented during printing onto the mandrel. 

### 2.2. Optical Microscopy Images

All optical microscopy images were imaged using a EuroMex Trinocular microscope with a PixeLink PL-B771F monochrome camera attached at magnifications varying from 0.7× to 4.5× and LED and incandescent illumination on a black or white background. Calibration of scalebars on images was then done using ImageJ version 1.49 s and a ruler.

### 2.3. Scanning Electron Microscopy Micrographs

The surface morphology of the 3D Printed samples was studied via Scanning Electron Microscopy (SEM). The samples were suck on a carbon stick pad on 8 mm or 12 mm diameter aluminium stubs and gold coated for 2 min (approx. 20 nm of gold coating) using an Edwards S150B sputter coater. SEM micrographs were collected using an Inspect™ F scanning electron microscope (FEI Company, Hillsboro, OR, USA) operating in secondary electron mode between 3–5 KeV and a spot size of 3.5–4. 

### 2.4. Tensile Testing

Tensile testing of the 3D printed ASTM standards was performed to evaluate the mechanical properties of the commercial PLA/PHA ColorFabb filament used in this study. The test was carried out using a Multitest 2.5-dV mechanical testing platform from Mecmesin equipped with a 1000 N load cell. The specimens were subjected to increasing load until failure by applying a deformation rate of 6 mm/min. The assay was carried out at room temperature using an ASTM D638 Type V standard CAD file with a gauge length 9.53 mm, width 3.18 mm and a thickness of 3.5 mm. This model was sliced with a wall thickness of 1.6 mm and layer height of 0.1 mm setting and printed at 215 °C with a 0.4 mm nozzle and printing speed of 12 m/s. Prior to the test, the thickness, and the width of each specimen in different areas was measured using a stainless-steel digital calliper and taken into account for the determination of the cross-sectional area. The data were acquired and analysed using Vector Pro software. Young’s modulus (E), Ultimate tensile strength (σ_u_) and Elongation at break (ε_u_) were calculated for each sample and averaged over *n* = 7.

### 2.5. Differential Scanning Calorimetry

Differential Scanning Calorimetry (DSC) was carried out using a TA (Thermal Analysis) instruments model Q20 DSC with an attached chiller unit. A small sample of the polymer (2.3 mg) was loaded into the machine and kept under an inert nitrogen atmosphere throughout the experiment. DSC measurements were carried out at heating and cooling rates of 10 °C/min. A heating cooling and heating program was used to obtain the melting temperature, *T_m_*, and glass transition temperature, *T_g_* of the samples. The sample was initially cooled to −80 °C followed by heating to 200 °C and then again cooled down to −80 °C. A final heating cycle was then performed to 200 °C. The *T_g_* was taken by midpoint fitting to the second heating cycle.

### 2.6. Fourier Transform Infrared Spectroscopy

FTIR measurements were carried out using a Thermo Scientific Nicolet iS5—FTIR Spectrophotometer. The printed sample was loaded onto the machine and firmly placed against the detector. Several scans were run and the ones with the best signal to noise ratio was chosen to do the analysis. All scans were confirmed to be similar prior to this. Absorbance spectra were registered between 400 and 4000 cm^−1^, with a resolution of 4 cm^−1^ and 45 scans. 

## 3. Results and Discussion

The 3D printing process requires the generation of a g-code file of the structure to be printed. This is obtained by means of a slicing software that converts 3D model files into slices of g-code that controls the motion and material feed as well as temperatures of the 3D printer. It is important to note that these software packages have been designed to optimally slice models for prototype development where the main objective is to have the outer-most layer of a model well defined. Therefore, the slicing software has two main functions when selecting parameters: the parameters for the wall features (visible to the observer’s eye) and the infill. Printhead motion has been optimised for infill patterns, where regular patterns included are rectilinear, honeycomb, concentric etc. Most 3D printing studies to date appear to use these functions as a method of generating structures with a given porosity, however this means that the user is limited to the exact design available in the slicing software, drastically limiting the advantages of printing. It is important to note that the major benefit in this work lies in that for the predefined infill patterns, printhead movement has been optimised to avoid crossing walls, therefore ensuring good definition of the infill structure. 

However, in the case of healthcare applications it is vitally important to have full control of the printed structure down to the smallest of features possible to mimic natural tissue, therefore here we take the approach of fully designing the final printed structures in 3D CAD avoiding the use of infill patterns for high resolution fine structures. 

For our models that are based on CT scans and have a more solid structure however the infill is still considered, and we chose it in a way to have a solid object. This was of interest for the grooved NGC, the mandible and tooth implant models. 

When designing models where the printer simply prints the walls it is therefore extremely important to ensure that the files are tuned towards the desired printhead nozzle diameter size, as otherwise the software will not detect features, and errors in the slicing of the model will occur. We found that it was advantageous to select a feature that is ever so slightly bigger than the nozzle size to ensure accurate picking up of features in the software. For example, for printing a 0.4 mm line, the model should have 0.39 mm line width, or the nozzle size selected in the slicing software parameters should be 0.41 mm in diameter for better slicing results. Hence, the critical values when slicing these types of models are: the nozzle size, shell thickness, and layer height, where the latter needs to be a multiple of the structure height to ensure correct slicing. This means that for ‘logs/struts’ that are e.g., 0.4 mm high a layer height of either 0.4 mm or 0.2 mm is appropriate, this can be further tuned by altering the material flow to avoid too little or excessive material flow. Finally, the shell thickness was considered to be the width of the nozzle diameter for our experiments. Normally the shell thickness would determinate how thick the outer layer is and where the infill pattern would start, however in our case the entire design is governed by the shell thickness and thus this needs to be tuned correctly to the smallest model feature lines and nozzle diameter which should all be equivalent. 

### 3.1. Printing of Tissue Repair Patches 

**Figure 2:**
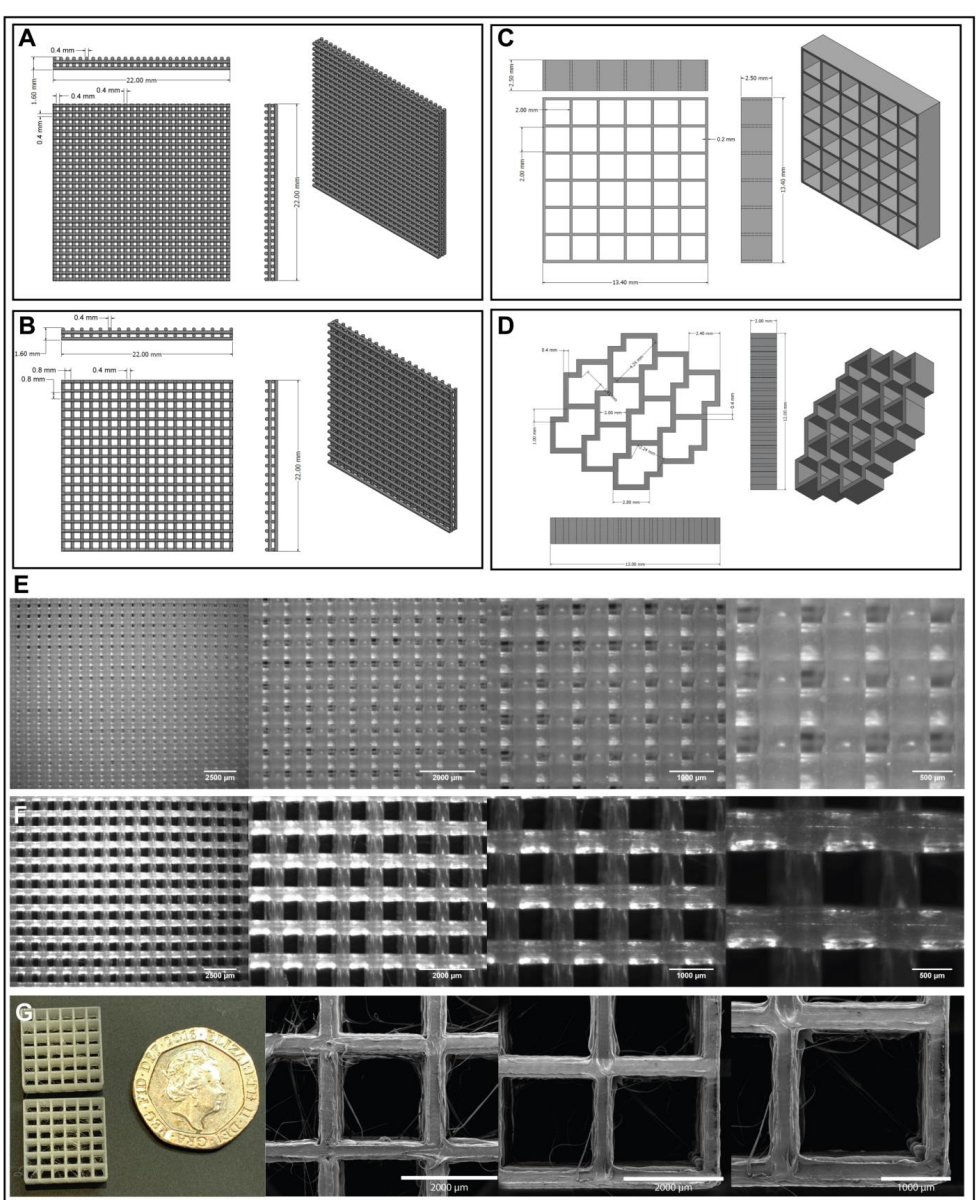
CAD designs of a variety of tissue repair patches: (**A**) Woodpile design with 0.4 mm strut diameter and 0.4 mm pore size, (**B**) Woodpile design with 0.4 mm strut diameter and 0.8 mm pore size, (**C**) Grid design with 0.2 mm strut diameter and 2 mm pore size, (**D**) Accordion design with 0.4 mm strut diameter and pores made by overlapping two squares each of 2 mm diameter. (**E**) Woodpile 4 layers 0.4 mm nozzle 2 mm/s print speed, 0.4 mm gaps, (**F**) Woodpile 4 layers 0.4 mm nozzle 2 mm/s print speed, 0.8 mm gaps, (**G**) Photograph and SEM micrographs of the grid design with 0.2 mm struts printed with a 0.2 mm nozzle at 2 mm/s print speed.

The patches were designed in Fusion 360. Three major designs were developed: woodpile structures (Figure 2A,B), consisting of logs layered on top of each other at 90 degree angles, grids (Figure 2C) with pore sizes of 2 mm, and an accordion structure as depicted in Figure 2D. These models were then converted to *.stl files and sliced in Creality Slicer 1.2.3 (for woodpiles and grids) and Ultimaker Cura 4.9.1 (for accordion) and consequently printed with a Creality Ender 5 at 215 °C, 60 °C bed temperature, a print speed of 2 mm/s, and nozzle diameters of 0.4 mm and 0.2 mm, respectively. 

The resulting 3D printed patches showed good definition and reproducibility as can be seen in Figure 2E,F. When printing more complex patterns such as the grids and the accordion structure the motion of the printhead was not optimised for the model and stringing could be observed in some cases (Figure 2F). It was possible to minimise the stringing by lifting the printhead during motion, however, to fully avoid this it is most likely necessary to manually modify the g code to avoid unnecessary moves and optimise the travel to get a continuous print.

The tissue repair patches designed here were based on previous research into cardiac patches [17,38,39,40,41,42,43,45,125,126,127,128,129,130,131,132,133]. The four designs were developed for printing as a proof of concept to assess optimal patch design and technology limitations. We note that for the use of cardiac patches flexibility is key and the PLA/PHA blend considered here does not provide the ideal mechanical properties. The constant contraction and relaxation of myocardial tissue requires an elastomeric material, and therefore our future work will consider an mcl-PHA (currently not available as a 3D printer filament). An ideal material would match the Young’s modulus of native human heart tissue, which lies between 0.02–0.05 MPa [134], and have a high elongation at break and tensile strength.

Engelmayr et al. (2008) [44] reported the production of a honeycomb structure grid design and put their research focus on creating a structure that when seeded with cells would produce an anisotropic cardiac patch. They initially used a poly(glycerol sebacate), PGS, scaffold of ~250 µm thickness as modelling analyses had shown this to mitigate mass transport limitations through PGS, however they acknowledge the problem that this is not a sufficient thickness for the reconstruction of full-thickness myocardial tissue. In an initial attempt to address this issue the researchers produced a bilaminar scaffold of ~400 µm and upon culture with neonatal rat heart cells found this produced a multi-layered tissue structure, however they suggest that this may require perfusion in order to maintain cell viability. A thickness of 2 mm has been proposed in the grid design cardiac patches described here and this is significantly thicker than that used by Engelmayr et al. [44], however the incorporation of endothelial cells to encourage vascularisation could mitigate the issues of oxygen and nutrient perfusion through the patch. The accordion-like honeycomb grid design was shown by Engelmayr et al. [44] to encourage directional alignment of heart cells and thus enabled them to create an anisotropic patch. Therefore, an accordion-like design was also designed here as a 3D printing proof of concept, however its ability to align cardiomyocytes will be assessed in future cell studies.

In another study by Gaetani et al. (2015) [41], human cardiac-derived progenitor cells in a gelatin/hyaluronic acid blend were 3D printed in rows like that of the woodpile designs described here. Each row was spaced 2.5 mm from the next, similar to the woodpiles, with a total width of 20 mm, just slightly smaller than the 22 mm width of the woodpiles. The matrices produced in this study were tested in vivo in a mouse model and found to reduce adverse remodelling of the myocardium and the preservation of cardiac performance. Differences arise between this design and the woodpiles, where in the Gaetani study [41] they incorporate the cells into the rows, however the woodpiles involve alternate logs of PHA and alginate encapsulated cells. They also had an overall thickness of 400 µm compared to the proposed 800–1600 µm of the woodpiles, however the incorporation of endothelial cells could overcome perfusion issues due to patch thickness, as mentioned previously.

Considerations must also be made in the future study of these cardiac patches—their purpose is for potential use in human patients, and therefore patches large enough for this purpose should be tested in vitro, however in vivo studies may take place in smaller animal models, hence, the size of the patch must be reduced relative to the size of the heart. These designs offer detailed structures which have not been the focus of most of the previous cardiac patch research, including Engelmayr [44] and Gaetani [41]. Key differences between the aforementioned studies and our work lie in the design of the patches as well as the material used, where a key future aim will look to using a PHA of optimal mechanical properties that closely match those of the human myocardium. Differences in dimensions include the thickness of the patch, with thicker designs proposed in this paper than in previous studies. However, these thicker patches must be tested with cells to assess their viability.

This study shows the ability of these cardiac patch designs to be created with a 3D printing processing technique, providing a proof of concept that will allow using other suitable materials for FDM 3D printing. Future studies will also look into the co-printing of hydrogel encapsulated cells to see how cells behave when cultured within these constructs, and to ensure their viability.

### 3.2. Design of Patient Specific Mandible Bone Repair via CT Scan and 3D Printing

#### 3.2.1. D model Generation from Real Patient CT Scan Data

**Figure 3:**
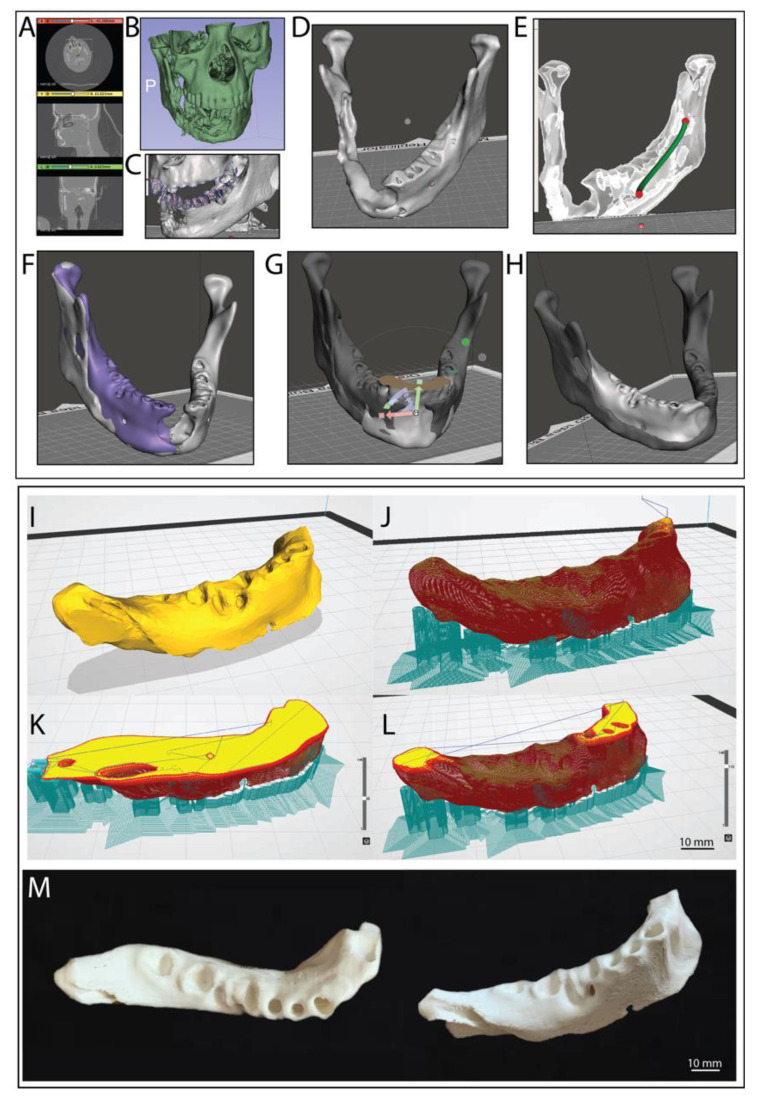
(**A**) CT scan of skull in the process of being cropped in order to isolate jaw; (**B**) segmented CT scan converted into a 3D model; (**C**) removing teeth and any joining surfaces between the skull and jaw; (**D**) Isolating jaw from skull via inverse election and converting the mesh to a solid; (**E**) Adding channel in order to preserve and define mandibular nerve channel; (**F**) Duplicating and mirroring of healthy side in order to replace missing jaw; (**G**) Filling gap in front using a CT scan of a healthy jaw; (**H**) Final implant after sculpting and smoothing; (**I**–**L**) Mandible Implant structure as seen in the Ender slicing software shown as different height regions, (**M**) 3D printed structure printed with a 0.2 mm nozzle at 215 °C.

In the pursuit of patient specific implants for replacement of repair we sourced a CT scan of a patient suffering from mandibular cancer from Embodi 3D^®^. The CT file was then imported into slicer 3D to isolate the desired tissue and ultimately convert it into a 3D mesh STL file. Our work involved initially cropping the image using the crop volume function to only leave the area of interest, in this case the mandible (Figure 3A). The tissue of interest was then isolated using the threshold effect. Here, a threshold range of 400 to 1500 appeared to give best results for the bone structure. It is important to note that this process will involve slightly different thresholds for every image as represented densities will differ from patient and machine. After applying the threshold, a model could be created using the make model effect (Figure 3B). The model was then exported as an *.stl file and imported into Autodesk Meshmixer to undergo further mesh editing and remove any 3D model defects.

Here, it was necessary to separate the mandible from the rest of the skull; this was achieved by using the brush select tool, to delete any joining faces between the jaw and skull (Figure 3C). Once the jaw was no longer connected to the rest of the skull, the jaw was selected and then, using the inverse selection tool, the rest of the skull could be removed (Figure 3D). After further mesh editing the mesh was turned into a ‘solid’ using the make solid function. Tools such as “shrink”, “smooth”, “flatten”, and “bubble smooth” tools were used to remodel the implant for an easier implant to jaw fit as well as repairing holes in the model which would cause issues during slicing for 3D printing (Figure 3D). Finally, a channel was added to preserve the mandibular nerve using the add tube tool (Figure 3E). 

#### 3.2.2. Removal of Unhealthy Tissue and Reconstruction Work

As in our case we have CT data from a patient with a severe cancer damage to the mandible structure and no precondition CT scan was available we attempted to reconstruct the lost/damaged tissue via general mirroring of the mandible from the healthy tissue to get the best possible approximation from the patients’ specifics. To achieve this the jaw was split down the middle using the splitting tool. The healthy side was then duplicated and mirrored making sure it was aligned to the original jaw structure as close to as possible (Figure 3F). The Boolean subtraction tool was then used to remove the cancerous region from the mirrored region creating an implant surface that matched the target region perfectly (Figure 3F (purple)). Once subtracted the implant was sculpted and edited to ensure the implant can be fitted with minimal difficulty during surgery. The final process involved filling in the front gap not covered by the healthy jaw copy, in this case a different CT scan was used of another person’s healthy jaw with similar physical structure to retain anatomical accuracy (Figure 3G). The new jaw was sliced, as previously described, only leaving the target area behind. The new component was aligned and once again using the subtract and union tools to modify the fit and join it to the implant (Figure 3H). Finally, the entire implant was once again sculpted to ensure a smooth join between the new added section and improve the overall fit of the implant (Figure 3H). The final model was then exported as an *.stl CAD file and sliced in Creality Slicer 1.2.3 (powered by the Cura engine) and printed on a Creality Ender 5 FDM printer. The jaw implant was printed with ColorFabb’s natural PLA/PHA filament, a layer height of 0.12 mm, 80 percent cubic infill, print speed of 20 mm/s and an overhang support up to $30^{{}^{\circ}}$. Figure 3I–L shows the finished model after slicing, where the red area indicates the wall thickness and yellow the infill sections. The turquoise sections show support material for overhangs, which could be easily removed after printing. The final printed structure is shown in Figure 3M. Printing with PLA/PHA filament and a 0.2 mm nozzle diameter resulted in very well-defined structural formation including the nerve channel and recesses for teeth. This study gives an excellent proof of concept, providing a pathway to fully patient specific implants based on FDM printing and PHAs as a way forward in regenerative medicine. Furthermore, there is a potential to combine this together with the following design of custom-made tooth implants that could be incorporated with this. Or even potentially printing as one structure if required.

The 3D printed mandibular implant was designed with the patient’s anatomy in mind, the implants aim was to reduce the change in appearance and function to the patient and instead identically replace what they lost during their mandibular resection. the materials used here give the implant biodegradability and biocompatibility properties allowing for the patient’s cells to be seeded into the construct and slowly replacing it to aim at a full reconstruction for the patient’s jaw which will hopefully be possible in the future. However, it would be possible to use the same process and non-biodegradable materials for implants that are required not to degrade or be replaced by natural tissue over time. The implants intention also relied on rapid design and manufacture; In a clinical setting this would include the process of a patient coming into a hospital with an injury, receiving a CT scan, which is then modelled, and 3D printed on site and implanted all in a day or two, vastly reducing waiting times. Often a lot of time is spent remodelling constructs during surgery as off the shelf do not always fit properly and the approach here will improve both the form and function of the implant. When looking at titanium mandibular implant, which are commonly used such as mentioned in the introduction they achieve to preserve the patients’ facial structure, however, they fall short in areas such as manufacture time, preservation of patient specific anatomy and the preservation of the bone tissue in the jaw. Furthermore, they fail to preserve structures such as the mandibular nerve channel, which on the other hand can be done when 3D printing with biodegradable materials as well as being ultimately cheaper to manufacture. 

### 3.3. Designing of Custom-Made 3D Printed Tooth Implants from Real CT Scans

**Figure 4:**
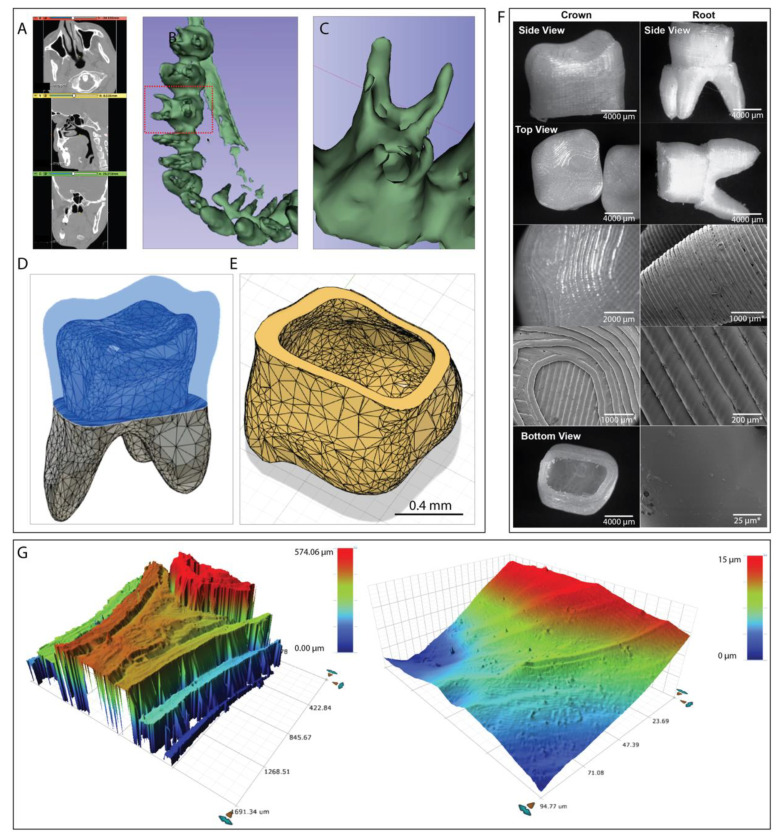
Design process of producing patient specific tooth implants via CT scan data. (**A**) Overview of CT scan area from different views; (**B**) Isolated view after picking threshold for a specific tissue density which emphasises the soft dental tissue area i.e., root of the teeth. (**C**) enlargement of the desired tooth for implant production. (**D**) representation of the root of the tooth after smoothing and repairing holes in the surface faces of the *.stl file with mesh mixer, with the crown region removed (blue) (**E**) CAD representation of the crown, fitting on top of the previous root model. (**F**) optical microscopy images of the 3D printed tooth implants Crown and Root. Scale bars marked with an asterisk (*) are SEM micrographs of the 3D printed tooth Implant, Crown and Root. (**G**) Profiler microscopy images of the 3D printed molar tooth surface.

Tooth implants are regularly needed by patients be it due to trauma, illness or decay. For the implants designed here it is plausible to also incorporate them together with a mandible replacement procedure, as described above, which could all be done via 3D printing either from the same material or different materials in a multi-material approach. For the generation of the tooth implant model we used a similar approach to creating the mandible. The generation of the patient specific tooth implant *.stl model file was extracted in a similar way as described in the previous section (Section 3.2). In our case the CT scan data was chosen from the same patient as the previous mandible implant (Figure 4A). However, in this case the tooth region was isolated, and the threshold picked to focus on the tissue density representing the teeth and roots (Figure 4B,C). The model was then split into two parts at the gumline to create a crown model file and a root file which both fitted into each other (Figure 4D,E). The two models were imported into Autodesk Meshmixer and post processed as previously described followed by surface smoothing. It is important to recognise when thresholding a CT scan the resulting shapes need some degree of manual refinement to ensure correct slicing and printability. For this task we found Meshmixer an excellent tool, enabling us to fill in any holes within the 3D model structure obtained from the CT scan. Furthermore, if certain areas were missing the surface was extrapolated accordingly to fill in the gaps. For the crown of the tooth implant an estimated thickness of 400 µm, which was digitally cut from the total tooth, was considered to be an acceptable approximation for this proof-of-concept work (Figure 4D,E). For this work the root and crown were printed separately as in the final application the materials would be different and therefore printed with a multiple extruder printer. The model was then sliced for printing and then printed with an 80% infill selected with a 0.2 mm nozzle and a layer height of 0.1 mm and 215 °C at a print speed of 2 mm/s, this resulted in a solid model with no noticeable gaps. As can be seen from Figure 4F both the tooth and the root resulted in good implant models, with well-defined features and a relatively smooth molar surface, representative of the nozzle size used. It is important to note that support structures were necessary to ensure proper bed adhesion and structure build up which did mean that some manual removal of support structures was necessary after printing. For the crown of the tooth we believe a non-biodegradable non-toxic material would be useful such as ABS, this could then be further post processed with solvents to give an entirely smooth surface after printing. The microscope and SEM images (Figure 4F) clearly show a definition of structures being printed resulting in a surface that was both very smooth along the printed lines but contains grooves between each printed line. Even though this structure might not be a perfect mimicry of natural tooth surfaces, the natural tooth surface also does contain grooves, so this will not to be a drawback for this type of tooth implant. It is possible to post-process the material in for e.g., in a chloroform atmosphere to allow the surface to become even smoother if desired. Increasing the printing temperature could also create shallower grooves, should these be considered too deep, however it is important to note that fidelity might suffer if the temperature is increased too much. Profiler microscopy images shown in Figure 4G further confirm these features and in terms of the smooth component of the surface a surface roughness value of *Ra* = ~1.06 µm and *Rq* = ~1.3 µm was measured.

### 3.4. Osteochondral Tissue Repair

**Figure 5:**
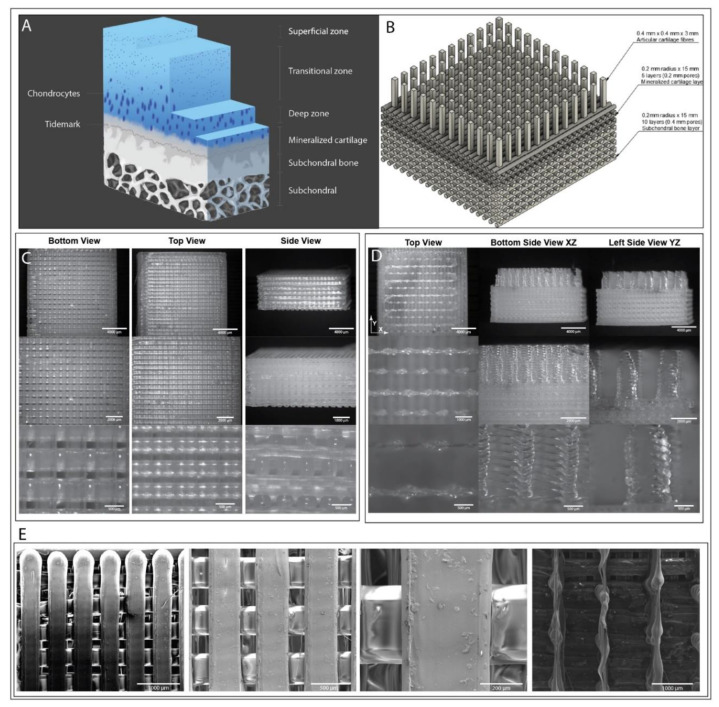
(**A**) Schematical representation of Osteochondral tissue; (**B**) Proposed CAD design of osteochondral repair plug; (**C**) double woodpile design of osteochondral plug as shown in B without pillars (printed with PLA/PHA ColorFabb natural filament and 0.4 mm nozzle at 2 mm/s and 215 °C; (**D**) Osteochondral Repair patch fully printed with 6 mm tall pillars. (**E**) SEM micrographs of the 3D Printed structure, 5 keV, 3.5 Spot size.

Degenerative diseases are of particular importance when looking at osteochondral tissue, causing much pain and suffering particularly in our ageing population. The repair of this tissue however is not trivial, which is why we aim to use the advantages of 3D printing to work towards a tissue repair solution to this problem. The osteochondral zone is shaped into three distinct layers capping one after the other to the articular cartilage surface within the joint. This zone begins with the normal subchondral bone tissue at the end of the bone itself, which transitions into a layer of chondrocyte-filled cartilage, which is interlaced with hydroxyapatite [135]. This mineralized or calcified cartilage layer initiates the change from the stiff bone tissue to the more gel-like cartilage cap and operates to modulate the changing mechanical properties as an energy transfer system [67,136]. Past the tidemark line (See Figure 5A), which distinguishes the calcified cartilage and the articular cartilage, the collagen fibres are initially orientated radially from the bone tissue to the cartilage surface but lose their orientation to become a parallel mesh lying flush to the surface of the cartilage [137,138]. Sparse populations of chondrocytes lie in patterns orienting to the direction of the fibres [139]. The lack of vascularisation and low cell density within the crucial cartilage layer that caps the bones is one of the main reasons why natural regeneration of this tissue is very limited [76,140]. Articular cartilage is both avascular and alymphatic, the cells within solely relying on their extracellular protein matrix for nutrient and waste transport throughout the tissue [141]. The osteochondral zone is a zonal transitioning interface between cartilage and bone and is found naturally within human joints [65]. It is composed of two major sections; a superficial articular cartilage layer and a deeper calcified layer which then transitions into the subchondral bone tissue [67]. Articular cartilage is typically around 2–3 mm thick and is interlaced with a dense matrix of collagen fibres of specific orientations, depending on their location within the layer. The topmost layer contains thin, ellipse-shaped fibres resting parallel to the surface of the cartilage, the middle zone contains randomly oriented fibres while the deep zone contains fibres oriented radially from the mineralized cartilage layer towards the surface [65]. 

The scaffold proposed here is a scaled model of this layer and attempts to retain native dimensions while being constrained to physical restrictions and current technological capabilities. Basic log piles form the basis of the bone section of the scaffold, which should be formed with a stiff polymer such as the one used here (PLA/PHA) or alternatively pure P(3HB). The side lengths of these pores were designed to be approximately 400 µm in side lengths, ideal bone cell diffusion, attachment and ingrowth with the extra space for eventual scaffold vascularisation based on results from previous studies on bone tissue engineering [142,143] (Figure 5B). The layer above, to represent the mineralized cartilage layer was selected to have a pore size of 200 µm to reflect the empirically determined range preferable for chondrocyte proliferation and fabrication of the extracellular matrix [144]. The final layer retains an inherent level of difficulty in construction for 3D printing. This layer consists of vertical aligned fibres, which would ideally be supported by a gel like matrix such as alginate. In native tissue, the fibres are very fine (10–300 nm in diameter) and close together to hold the tissue’s high-water content together to retain function [21,141]. 

For the final prototype we would envisage that the 3D printed scaffold would be topped with a randomly aligned electrospun or gyrospun fibrous mat, which would have fibre diameters of 100–150 nm, as has been performed in previous studies [15,145]. This is to mimic the topmost layer of the articular cartilage, where the fibres change direction to rest parallel to the surface of the articular cartilage, while maintaining the random fibre alignment in that plane [146]. 

Our printing results indicate that the lower subchondral bone layer and mineralised cartilage layer were very printable (Figure 5C,E). The porosity can be clearly seen from all angles including the sides of the structure and is very well defined throughout. The transition between the two different porosities is very clear and connectivity of the two sections adhered very well with each other with no delamination. The articular cartilage pillars however proved more challenging to print. Here, the difficulty was due to the layer-by-layer printing approach most printers use resulting in a print with stringing issues, where material was dragged across from one pillar to the next, see Figure 5D,E. To solve these issues a future option might be electrospinning aligned fibres for this section and combining it with the printed structure. The use of finer nozzles as they become available on the market will also help with higher definition of complex structures like these and finally using a printing approach that does not rely on layer-by-layer fabrication might also help. Furthermore, it is debatable if the stringing would have a negative impact on the final scaffold design in vitro as the stringing might actually give an advantage in terms of initial structural support during scaffold fabrication and initial cell seeding and would be expected to be re-absorbed much earlier than the thicker structures. Many currently accepted cartilage tissue engineering scaffolds use a biphasic model to represent the different articular cartilage and subchondral bone layers. However, the osteochondral zone is formed of three layers and models which have reflected this have delivered good experimental results of tidemark development and separated tissue growths [147]. The application of 3D printing in the fundamental production of complex multi-material scaffolds for cartilage engineering are prolific as the manipulative capabilities ensure that the scaffold can be made as per design, and materials can range from stiff synthetic polymers to biological-based hydrogels [148]. The scaffold model designed here was specifically created to replicate the organized structure within the endogenous tissues and can be redesigned to fit the parameters of patient-specific defects in the near future, paving the way for patient specific implants and the ability to utilise the patient’s own cells to ensure total compatibility. 

### 3.5. 3D Printed Nerve Guidance Conduits

Dependent on their location in the body, nerves can be very large such as the sciatic nerve or much smaller in dimension, this gives rise to different dimensional requirements when persuading nerve repair and regeneration. For our designed conduits we looked at creating a patient specific sciatic nerve conduit modelled from real patient CT data, as well as simple tube-based conduits suitable for peripheral nerve injuries. 

#### 3.5.1. Modelling Process of Sciatic Nerve from CT Scan

**Figure 6:**
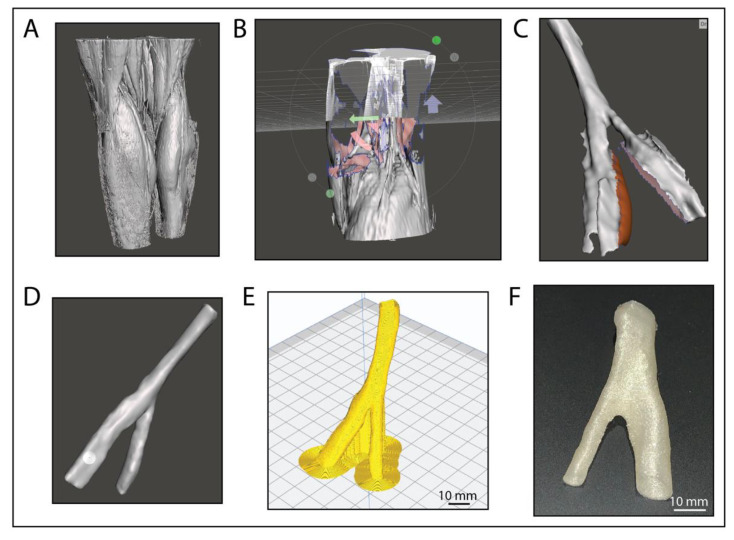
(**A**) Muscle and nervous tissue isolated in CT scan and converted to a 3D model; (**B**) Isolating sciatic nerve from surrounding soft tissue using selection and deletion tools; (**C**) Further isolation of the sciatic nerve and the removal of excess tissue; (**D**) Smoothing of sciatic nerve using sculpting tools and hollowing using the make hollow tool; (**E**) Slicing nerve conduit in slicer for 3D printing. (**F**) Photograph of the 3D Printed sciatic nerve conduit 0.2 mm nozzle diameter printed at 215 °C.

The nerve guidance conduit followed a similar procedure to the jaw implant. An appropriate CT scan file was sourced from Embodi 3D^®^ and imported into slicer 3D and the area of interest cropped, in this case the sciatic nerve (Figure 6A). In contrast to the previous implants, it was more challenging to isolate the sciatic nerve due to the lack of discrepancy between soft tissues. It was impossible to fully isolate the nerve and instead a compromise was made between retaining as much of the nerve tissue as possible, while also having the least amount of other soft tissues such as epithelial tissues, muscular tissue and lipids (Figure 6A). A threshold of −50 to 100 was deemed most appropriate for our CT data. The file was then imported into Meshmixer (Figure 6B) and the select and erase and fill tools were used to remove as much of the unwanted tissue as possible (Figure 6C). Once the nerve was isolated, the 3D model was further post processed as previously described and a final step was to make the nerve hollow using the hollow tool (Figure 6D), after this the nerve was sliced in Cura slicer (Figure 6E). The nerve was printed with ColorFabb’s white PLA/PHA filament, at a layer height of 0.12 mm, 20% cubic infill, print speed of 50 mm/s and overhang support up to $30^{{}^{\circ}}$ which resulted in a very fine well-defined print as shown in Figure 6G. This 3D printed nerve conduit addresses the lack of specificity and adaptability present by current nerve replacement techniques. The 3D printed sciatic nerve conduit not only stays true to the patient’s anatomy with regards to size and form, but also addresses complex nerve repairs like the splitting of a nerve into two channels, a structure which is demonstrated by the splitting of the sciatic nerve behind the patella. Where conventional conduits would require cutting and stitching in order to attain the same shape, the 3D printed conduit can be manufactured into any shape and yields the possibility for far more complex geometries than that of their electrospun counterparts. The nerve conduit also eliminates the need for a nerve autograft as the conduit channel is intended to act as a scaffold and allow for new cells to proliferate along the channel and reconnect the severed nerve endings.

#### 3.5.2. CAD Design of Neural Guide Conduits

**Figure 7:**
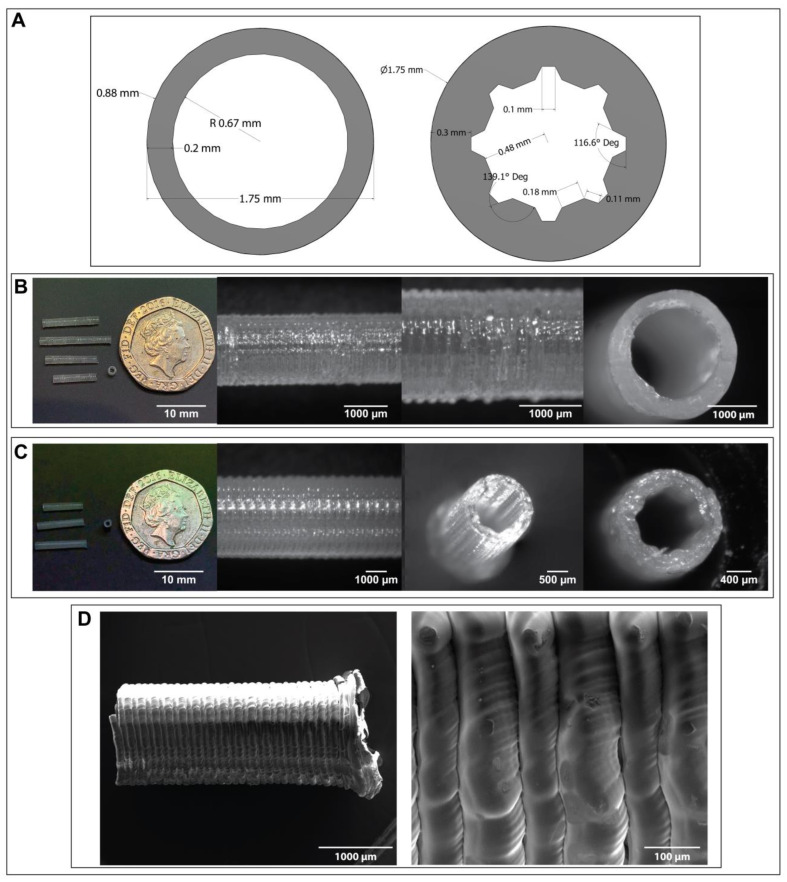
FDM-Printed Nerve Guidance Conduits with ColorFabb natural PLA/PHA filament on an Ender 5 3D Printer equipped with a 0.1 mm nozzle printed at 215 °C. (**A**) CAD design of hollow tube NGC (left), CAD design of grooved NGC (right), (**B**) 3D printed hollow tube NGC, (**C**) 3D printed grooved design NGC, printed with a 0.1 mm nozzle, (**D**) representative SEM micrograph of a NGC as depicted in (**A**–**C**), imaged at 3 KeV and a 4.0 spot size.

For smaller, more simplistic peripheral nerve injuries NGCs can be easily and directly designed with a CAD software, here we created two simple designs, namely a single lumen tube and a grooved tube with the dimensions as depicted in Figure 7A (right and left respectively), where the length of the construct can be chosen according to the implantable location. Several NGC designs to date have been reported, which can be categorized into 5 groups: (i) Hollow/non-porous design [92], (ii) grooved design [149,150], (iii) porous design [151,152], (iv) multi-channel design [153], and (v) NGCs with fillers [154,155,156]. Ideally, NGC designs should have topographical guides for aligning cell growth in a specific direction, so random or disordered structures should not present in conduit structures. 

Existing nerve conduits to date have proven inefficient over severe or larger gap injuries (exceeding 40 mm). This is attributed largely to their inability to mimic the biological characteristics of a native nerve tissue [157,158]. Designing prototypes that can meet the requirements of the native microenvironment of nerve tissues is the current focus of research. Research towards next-generation nerve tubes includes the search for suitable materials with sufficient biocompatibility and preferred biodegradability profiles, with tuneable physico-chemical properties that can result in constructs with required mechanical compliance. Further, the tubes are to be enriched with appropriate topographical and electrical cues, incorporation of luminal fillers and growth stimulants like Schwann cells, neurotrophic factors (nerve growth factors, glial-derived neurotrophic factors), extracellular matrix proteins, as well as suitable advanced fabrication techniques [159]. 

Several methods have been used traditionally to fabricate nerve tubes including, electrospinning, melt extrusion, crosslinking, gas foaming, physical film rolling, freeze drying, injection moulding, melting extrusion and braiding [160,161]. Common problems associated with these techniques are their inability to control porosity, difficult to reproduce, and non-defined multi-layered structure cut across them [161]. In recent years, three-dimensional (3D) printing is emerging as an advanced technique that changes the status quo across the biomedical engineering field with enhanced reproducibility and customizability. 3D printed scaffolds are reported to provide cells with the ideal interphase for anchorage, high surface area to volume ratio for attachment, porosity, and above all the microenvironment required to support cell growth, proliferation and regeneration sufficiently [162].

We find our 3D printing method reliably prints the two designs with high definition of the structures. The inner diameter and a wall thickness used were around 1.1 mm and 0.250 mm respectively for the single lumen hollow nerve conduit, are considered ideal dimensions [163]. Optical and SEM microscopy images can be seen in Figure 7B–D, where the filament can be clearly seen with smooth surfaces on the extruded areas. Literature has reported better nerve guidance due to grooves and therefore we consider that our grooved design should help enhance the directional regeneration for peripheral nerve injuries [164]. Here the ability to 3D Print PHAs as the main material provides the foundation of novel highly tuneable NGC designs for the future. For future improvement we would consider the printing of a blend of SCL and MCL PHAs to be advantageous to give the NGCs more flexibility for in vivo studies. 

### 3.6. Design of a Coronary Artery Repair for the Heart

The construct here was designed with the aim of being applied in the setting of the Fontan procedure for treatment of the Hypoplastic Left Heart Syndrome (HLHS). This is where a conduit is used to redirect blood from the inferior vena cava to the pulmonary artery, also known as a total Cavo pulmonary connection. The conduit here is specifically designed for use in the extracardiac Fontan procedure, where the conduit is placed external to the heart [165]. Along with its function as a conduit, we believe it would be ideal for a final design to act as a scaffold for the generation of a contractile cardiac tube with live cells act as a ventricle in the future this will depend on material properties used in a final structure to give flexibility for contraction of the cells. Our work here looks at a novel way of printing via a custom-built mandrel to achieve high-definition tube structures via CAD design and the use of our model commercial PLA/PHA ColorFabb filament. 

**Figure 8:**
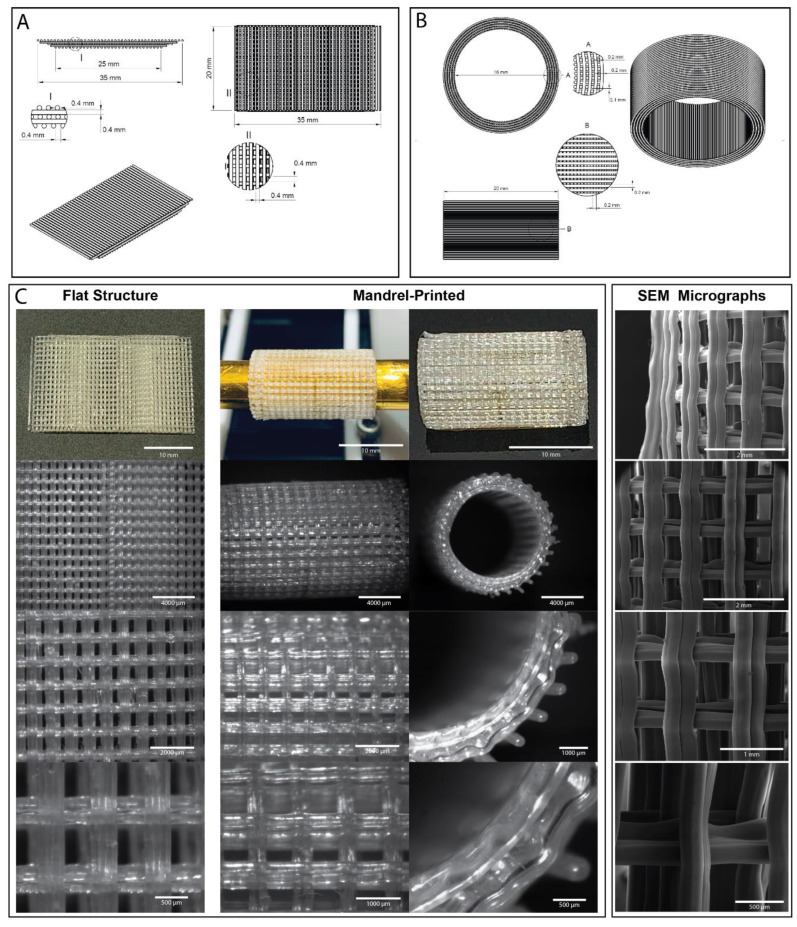
(**A**,**B**) CAD design for replacement Coronary Artery repair designed in Autodesk Fusion 360 (**A**) theoretical structure how the final design should look with ideal dimensions for application in a human. (**B**) flat designed structure with a strut diameter of 0.4 mm designed for printing on our custom built 8 mm (diameter) 3D printer mandrel attachment. (**C**) Single Ventricle printed on flat surface and printed on 8 mm (diameter) 2 cm long mandrel. 0.3 mm nozzle, printed at 215 °C.

The CAD design for the coronary artery repair was developed in Fusion 360. A CAD file for a flat woodpile model was created to be first printed on a flat surface (Figure 8A) to finetune printer settings and get the best possible print prior to printing on a custom-built novel 8 mm mandrel to be directly printed as a tubular shape. This produced a cylindrical model as identical in Figure 8B (however with smaller diameter) as a proof-of-concept work. For this type of print it was important to consider that each layer of the model would require different lengths due to the changing outer diameter as progression through each layer added the thickness of the respective layer. To calculate the number of cylinders on each layer containing those parallel to the long axis (Equation (1)) was used, where *N_perfr_* refers to the number of parallel fibre repeats, C the desired circumference *p* the pore size and *d_fibre_* the fibre diameter. The length of these cylinders was the height of the construct. Using the rectangular repeat tool, the calculated number of cylinders was produced. The parameter of distance was filled as half the desired circumference, as for accuracy a symmetrical repeat was used, ensuring equal numbers of cylinders either side of the centre. (Equation (2)) was used to calculate the number of cylinders on the layers containing fibres perpendicular to the long axis, where *N_pfr_* refers to the number of perpendicular fibre repeats, *h* the construct height. The cylinder length corresponded to the circumference of the desired layer. The rectangular repeat tool was used to generate the calculated number of cylinders across the distance of the conduit height. Strut diameter of the logs was chosen to be 0.4 mm for the proof-of-concept work.

(1) $$N_{perfr}=\frac{C}{p+d_{fibre}}$$

(2) $$N_{pfr}=\frac{h}{\left(p+d_{fibre}\right)}$$

We opted to produce a grid like structure to enable cells to fully penetrate the structure, this also gives a clear advantage of 3D Printing over other techniques such as dip coating which would not be able to replicate our structure. 

The ability to produce a dynamic implant for this procedure is vital as when this procedure is done on young individuals the body is still growing and it needs to be replaced later with a larger implant. Therefore, the future lies in fabricating a fully biocompatible structure that can adapt and grow. Undersized conduits of 14–16 mm for the Fontan procedure have been associated with an increased incidence of stenosis and energy loss with growth [166]. This stenosis is due to outgrowth of the graft by the patient, as the conduit is stretched when the patient ages and their heart grows in size. This is especially noted when they go through their teenage growth spurt due to this being over a relatively rapid period of time [167]. However, implantation of a larger size conduit for the patient to theoretically grow into in order to counterbalance this risk, does not work. Ideally an 18–20 mm diameter conduit would be the desired size for an adult, but this is far too large for the size of the patient at implantation [167], especially if it is considered that these surgeries are generally done between the ages of 18 months and 4 years [168]. Revision surgeries of the Fontan procedure were found to be done in this study at an average of 9 years from the initial procedure [169]. These may be done for a variety of reasons including obstruction of the conduit or failure of the Fontan circulation. As this conduit is designed to encourage growth of the patients own tissue to replace the temporary PHA construct, it gives a connection with growth potential. The conduit should grow at the same pace the patient does, giving the correct size to meet the patient’s cardiovascular needs. As the conduit is made of a bioresorbable PHA blend, it will naturally disintegrate into the body and not require removal. This also has the benefits of no requirement for removal, alongside a reduced need for revision procedures. Figure 8C shows optical and SEM microscopy images of the printed construct, as can be clearly seen, we observed a good definition of structure that was removable from the mandrel without destruction. The struts are smooth and well-formed, which is important especially on the inner side of the structure. Furthermore, this method gives a simple method of producing complex tube-like structures via CAD design and mandrel 3D printing. 

### 3.7. Design of 3D Printed Stents

**Figure 9:**
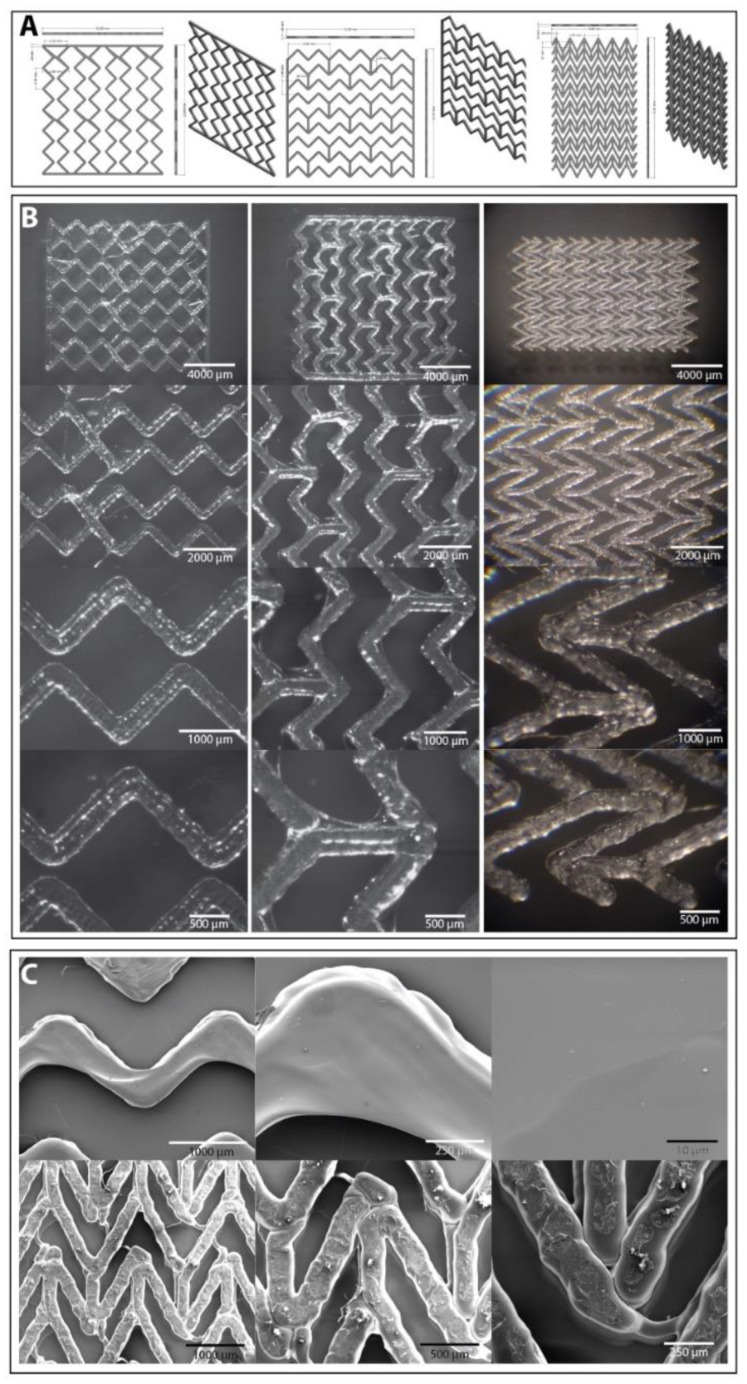
(**A**) CAD designs of three different polymer stents (flat design) with strut diameters of 0.2 mm. (**B**) Optical microscopy images of the three stent designs at different magnifications after 3D printing with a 0.15 mm nozzle at 215 °C; (**C**) representative SEM micrographs of the printed stent designs.

The Stent designs were designed in Fusion 360. Three designs were designed, which were modelled against currently available stent designs as closely as possible. Parameters were assumed as these designs are protected by IP and not readily available (see Figure 9A). For all stent designs strut diameters of 0.2 mm were chosen. The models were then converted to *.stl files and sliced in Creality Slicer 1.2.3 (for woodpiles and grids) and Ultimaker Cura 4.9.1 (for accordion) and consequently printed with a Creality Ender 5 at 215 °C, 60 °C bed temperature and a print speed of 2 mm/s and a nozzle diameter of 0.15 mm. Polymer stent designs mentioned in literature generally have thicker strut diameters compared to metal stents, namely of around 0.17 mm in diameter [107,170,171]. The average stent length according to literature was considered to be 12 mm in length [107,171]. Dependent on the blood vessel diameters of 3 mm, 3.5 mm and 4 mm are all considered of interest [171]. A key contributor in the development of biodegradable polymer stents is Abbott. The Absorb GT1 Bioresorbable Vascular Scaffold (BVS) is a temporary scaffold that will fully resorb over time [172]. According to their website this stent is around 24 mm in length with a varying diameter from 2.5 mm to 3.75 mm [172]. Our stent designs were based on a flat surface, however these can be in future be printed like the coronary artery repair in the previous section on a mandrel for a tube like design with appropriate mandrel diameters. We find our stent prints to be reproducible and with high fidelity. 

### 3.8. Material Characterisation of PHA/PLA ColorFabb Filament Blend

**Figure 10:**
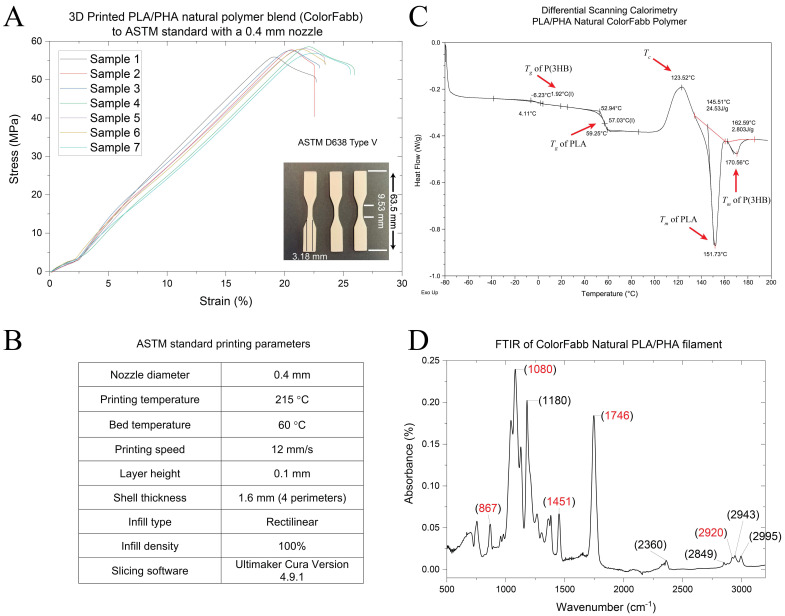
(**A**) Examination of the tensile properties of the ColorFabb PLA/PHA blend after printing ASTM 638 Type V standard samples (gauge length 9.53 mm, width 3.18 mm, thickness 3.5 mm) inset: example 3D printed samples, (**B**) Printing and slicing parameters used for the printing of the ASTM D638 Type V samples (1–7), (**C**) DSC of ColorFabb natural filament used in this study and fitted *T_g_* and *T_m_* values for PLA and P(3HB). (**D**) Fourier Transform Infrared spectrograms (FTIR) for 3D printed ColorFabb natural filament, peaks labelled with red numbers are representative of the PLA component of the polymer blend.

Six samples of the PLA/PHA natural ColorFabb filament blend used for all our structures were printed according to the ASTM D638 V standard (gauge length 9.53 mm, width 3.18 mm, thickness 3.5 mm). These were printed with a 0.4 mm nozzle (see printing parameters Figure 10B) and tested on a mechanical testing machine, which resulted in reproducible results as shown in Figure 10A. The initial irregularity in the data is due to stability issues at the very start of the testing. Reproducible results of all samples were found to be a Youngs Modulus of 180.7 ± 27 MPa, an elongation at break of 30.4 ± 2.1% and an ultimate tensile strength of 56.7 ± 0.9 MPa were measured. 

The Youngs modulus measured in this study appears to be ~6 fold lower than those obtained by Gonzalez et al. [173], however the elongation at break is ~10 fold higher, a possible reason for this could be due to different printing parameters. Also, the age of material and printing temperatures (ours 215 °C to theirs 200 °C) might also have an effect, for example we used 4 perimeter shells (1.6 mm) whereas they use only two. The method of tensile testing was also carried out at different speeds. In their study they use 20 mm/s and in our study, we use 6 mm/s, which we have found to alter mechanical properties of the printed polymers in the past. The authors also use a different ISO standard to ours (namely ISO 527). Both of these methods are standards accepted for thermoplastic polymers, however it is important to note that unlike mould casting, 3D printed structures will have properties dependent on nozzle size, filament thickness, adhesion of one filament strand to the next, printing temperature, infill methodology, peripheral wall thickness and layer height. In addition to this, material degradation during the printing process as well as material age of the thermoplastic polymers post printing will influence results. Our results when compared to the study by Gonzalez et al. of the same material, therefore confirm that it is important that mechanical properties of the actual printed structure should be measured for each study rather than relying on published values. Our data however does show that when the same material, slicing and printing settings are used, the reproducibility of the final structure is very high with 3D printing. Given these facts, it shows that for 3D printing it might be useful, in the future, to develop a standard based on not only on the model shape but also the printer slicing parameters, in order to ensure better reproducibility.

Further to this DSC measurements (Figure 10C) of the printed polymer blend concluded that the blend was not miscible and therefore resulted in two melting (*T_m_*) and glass transition temperatures (*T_g_*) that are characteristic of both materials. We found the *T_g_* of the PLA component in our blend to be ~57 °C and *T_m_* to be ~152 °C and the *T_g_* of the PHA to be ~1.9 °C and *T_m_* ~171 °C, which are similar to the known values for PLA and the PHA, P(3HB) respectively. Very similar values were obtained by Gonzalez et al. [173] for the same commercial filament. There is however one variation to their study where the exothermic crystallisation temperature, *T_c_*, measured was 141–142 °C, however for this study it was measured to be 123.52 °C which might be another reason for the differing mechanical properties. This difference might indicate a batch-to-batch variation from the supplier, this might be due to variation of the PHA component of the polymer blend either in molecular structure or also in percentage. As Gonzalez et al. [173] indicate, most likely the PHA component of the blend will have a positive influence on the crystallinity of the polymer blend. We also found that the P(3HB) content of the polymer blend degraded when the DSC was run higher than 220 °C, so our printing temperature range of 205–215 °C should be in acceptable parameters, especially as the polymer should only be heated a short while to this temperature as it passes through the Bowden extruder nozzle. Fourier Transform Infrared spectrograms (FTIR) data corresponds to data in literature for both PLA and P(3HB), where distinct peaks corresponding to -C-O-C- bond stretching (867 cm^−1^), -CH_3_ asymmetric vibrations (1080 cm^−1^), -CH vibrations (1451 cm^−1^), -C=O vibrations (1746 cm^−1^) and -CH_3_ symmetric vibrations (2920 cm^−1^) for PLA could be detected. In the case of P(3HB) bands corresponding to -CH_3_ asymmetric stretching vibrations are in the range of 3015–2955 cm^−1^ and 2885–2845 cm^−1^ for the -CH_2_ asymmetric stretching vibrations. The peaks at 2849 cm^−1^ and 2943 cm^−1^ indicate the crystalline state of P(3HB) and the peak at 2995 cm^−1^ arises from the amorphous phase (Figure 10D). Thus, the FTIR data of the polymer blend confirms the presence of PLA and P(3HB) [174]. Comparison of the FTIR data indicated that the PLA peak at 1746 cm^−1^ is slightly lower than that reported by Gonzalez et al. [173] where they find a peak at 1755–1760 cm^−1^ which then gradually becomes lower, as the polymer degrades in their polymer degradation study. This could have two explanations, namely a batch-to-batch variation from the material suppliers as well as a potential that our initial material was slightly more degraded, prior to printing, due to age or related to changes in the filament making conditions. 

The mechanical properties measured indicate that this polymer blend is suitable for applications such as stent design and potentially the lower layers of the osteochondral structure we designed. There is also some potential for this blend to be used for NGCs and tooth implants and the mandible work demonstrated here. For the other applications, a softer elastomeric polymer or polymer blend will need to be used that encompasses an elastomeric medium chain length PHA (MCL-PHA).

## 4. Conclusions

In this work we have successfully demonstrated that commercially available PLA/PHA filaments for FDM printers can be used to create very intricate and fine structures for a large variety of biomedical applications as proof on concept studies including but not limited to tissue repair patches, bone repair, tooth implants, osteochondral tissue repair, nerve conduits, stents and heart ventricles. In order to achieve this, we discussed the importance of correct model designs and selection of slicing software parameters, where nozzle diameter, layer height and shell thickness parameters are of key importance when generating usable g-code for intricate designs. Unlike many other 3D printing studies, we do not use the use the infill pattern and density settings as standard to create our scaffolds (available in slicing software) as these are hugely limiting for fine structural designs. We find that for good slicing conditions layer height, shell thickness and nozzle diameter parameters should be identical, where it was seen advantageous for some model designs to have a nozzle diameter ever so slightly larger than the thinnest line thickness of the models to ensure correct g-code values. We print structures with nozzle diameters ranging from 0.1 mm to 0.4 mm dependent on the application. We have been able to print structures down to resolutions of 100 µm with high reproducibility and accuracy for stent and NGC designs. 

In addition, we generated patient specific custom models for bone, tooth and nerve implants developed from real patient CT scans remodel missing areas, due to e.g., cancer growth or traumatic injury, and successfully print perfect fit replacement implants to repair the missing sections. Furthermore, we show the benefits of the bottom-up approach of printing a mandible bone implant rather than the traditional machining of the construct CNC methods as it is possible to ensure the nerve channel is preserved in the implant, which is valuable for future bone scaffolds that will hopefully eventually be fully regenerated by cells in the patient for full sensory functionality. 

Finally, by using one of the first commercially available PLA/PHA based filaments we pave the way to use PHAs as a desirable thermoplastic polymer for 3D printing biomedical scaffolds. Our material characterisation of the commercial ColorFabb PLA/PHA filament suggest that its tensile properties might be very useful to applications such as 3D printed stent design, NGCs, and bone scaffolds. We believe that the PHA used in the ColorFabb PLA/PHA filament corresponds to material characteristics of P(3HB) along with the PLA characteristics, confirmed via DSC and FTIR.

We see the next step now to implement these proof-of-concept structures to be used in vitro with specific cell lines for each application and then future in vivo experiments. As each medical application requires specific mechanical properties of the structures these can in future be tailored via both structure design as well as novel PHA material blends that incorporate both MCL and SCL PHAs paving the way for more versatile applications to mimic each environment including altering physical properties such as adhesion, which will help with cell attachment. We have successfully demonstrated that it is possible to print high quality structures with an aim at biomedical applications with low-cost off-the-shelf FDM extrusion printers such as the Ender 5 used here, which will allow rapid adaptation and implementation of this technology in the near future. This will give developing countries excellent opportunities not to be left out in this exciting medical development and on-site rapid development of patient specific implants, which will ultimately expedite patient recovery with an eco-friendly technology. Finally, we believe that developing FDM filaments based entirely on PHAs would be an excellent step in paving the way for widespread use of these biocompatible, biodegradable and sustainable polymers for biomedical applications as well as any other applications that utilise 3D printing fabrication methods.

## Data Availability

Data is available on request from the authors.

## Abbreviations

ABS	Acrylonitrile butadiene styrene
AFM	Atomic Force Microscopy
AM	Additive manufacturing
CAD	Computer aided design
CAD	Coronary artery disease
CAWS	Computer-aided wet-spinning
CT	Computed tomography
CVD	Cardiovascular disease
DSC	Differential scanning calorimetry
ePTFE	Expanded poly(tetrafluoroethylene)
FDM	Fused deposition modelling
FTIR	Fourier transform infrared spectroscopy
LBL	Layer by layer
MCL-PHA	Medium chain length PHA
MRI	Magnetic resonance imaging
NGC	Nerve guidance conduit
P(3HB)	Poly(3-hydroxybutyrate)
P(3HB-*co*-3HHx)	Poly(3-hydroxybutyrate-co-3-hydroxyhexanoate)
P(3HO)	Poly(3-hydroxyoctanoate)
P(4HB)	Poly(4-hydroxybutyrate)
PCL	Polycaprolactone
PDLLA	Poly(DL) lactic acid
PEG	Poly(ethylene glycol)
PGS	Poly(glycerol sebacate)
PHA	Polyhydroxyalkanoate
PLA	Polylactic acid
PLGA	Poly(lactic acid-co-glycolic acid)
PLLA	Poly(L-lactic acid)
PNI	Peripheral nerve injury
PNS	Peripheral nerve system
PTCA	Percutaneous transluminal coronary angioplasty
PVA	Poly vinyl alcohol
SCL-PHA	Short chain length PHA
SEM	Scanning Electron Microscope
SLS	Selective laser sintering
